# Screening Ammonium‐Based Cationic Additives to Regulate Interfacial Chemistry for Aqueous Ultra‐Stable Zn Metal Anode

**DOI:** 10.1002/advs.202407102

**Published:** 2024-09-28

**Authors:** Leilei Zheng, Huihua Li, Mingbo Gao, Keer Huang, Jian Wang, Long Su, Lei Li, Hongzhen Lin, Xinpei Gao, Zhengqing Liu, Huang Zhang

**Affiliations:** ^1^ Key Laboratory of Engineering Dielectric and Applications (Ministry of Education), School of Electrical and Electronic Engineering Harbin University of Science and Technology Harbin 150080 P. R. China; ^2^ Institute of Flexible Electronics Northwestern Polytechnical University Xi'an 710072 P. R. China; ^3^ i‐lab & CAS Key Laboratory of Nanophotonic Materials and Devices, Suzhou Institute of Nano‐Tech and Nano‐Bionics Chinese Academy of Sciences Suzhou 215123 P. R. China; ^4^ Helmholtz Institute Ulm (HIU) D‐89081 Ulm Germany; ^5^ Karlsruhe Institute of Technology (KIT) D‐76021 Karlsruhe Germany; ^6^ Key Laboratory of Colloid and Interface Chemistry (Ministry of Education) Shandong University Jinan 250100 P. R. China; ^7^ Key Laboratory of Advanced Materials in Tropical Island Resources (Ministry of Education), School of Chemistry and Chemical Engineering Hainan University Haikou 570228 P. R. China

**Keywords:** cationic electrolyte additives, interfacial chemistry, solvation shell structure, theoretical simulation, zinc metal anodes

## Abstract

The interfacial dynamics and chemistry at the electrolyte/metal interface, particularly the formation of an adsorption interphase, is paramount in dictating the reversibility of Zn metal deposition and dissolution processes in battery systems. Herein, a series of different cationic ammonium‐based electrolyte additives are screened that effectively modulate the interfacial chemistry of zinc anodes in aqueous electrolytes, significantly improving the reversibility of Zn metal plating/stripping processes. As initially comprehensive investigation by combining theoretical calculation and molecular dynamic simulation, the tetramethylammonium cation, with its specific molecular structure and charge distribution, is identified as pivotal in mediating the Zn(H_2_O)_6_^2+^ solvation shell structure at the electrode/electrolyte interface and shows the strong resistance against electrolyte corrosion as revealed by X‐ray and optical measurements. As a result, the Zn||Zn symmetric cell with optimal electrolyte lasts for over 4400 h of stable plating/stripping behaviors, and the Zn||Cu asymmetric cell stabilizes for 2100 cycles with an average Coulombic efficiency of 99.8%, which is much better than the‐state‐of‐art progress. Consequently, full‐cells coupled with various cathodes showcase improved electrochemical performance, displaying high capacity‐retention and low self‐discharge behaviors. These findings offer essential insights of cationic additives in ameliorating zinc anode performance.

## Graphical abstract

The interfacial chemistry on Zn anode can be regulated by cationic additive strategy, proving as an efficient approach suppressing side reaction and maintaining Zn deposition behavior. As a proof‐of‐concept, the quaternary methylammonium cationic additive endows the Zn anode with a ultralong cycle life and high Coulombic efficiency in aqueous batteries.

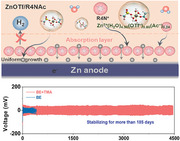

## 1 Introduction

Electrochemical energy storage (EES) plays a pivotal role in harnessing renewable energy for grid‐scale electricity, ensuring the dependability of energy supply.^[^ ^1^ ^]^ Among all the available EES technologies, rechargeable aqueous zinc metal batteries (AZMBs) stand out as a potential candidate for large‐scale energy storage, featured by their intrinsically high safety, low cost, and environmental friendliness.^[^ ^2^ ^]^ However, their practical applications are impeded by suboptimal cycling stability and low Coulombic efficiency (CE), mainly due to the challenges associated with the irreversible zinc metal anode, including dendrite formation and parasitic side reactions such as hydrogen evolution reactions.^[^ ^3^ ^]^

Toward inhibiting dendrite growth of uniformizing Zn ion flux, several strategies have been proposed to improve the reversibility of Zn anode in AZMBs,^[^ ^4^ ^]^ such as interfacial designs,^[^ ^5^ ^]^ electrolyte engineering,^[^ ^6^ ^]^ and surface structural modification of Zn anode.^[^ ^7^ ^]^ Among them, electrolyte additive, as an efficient electrolyte engineering approach, has been widely applied and shown great success in batteries, which can efficiently inhibit the dendrite growth of metal anode and stabilize the interface during electrochemical cycling. For example, the application of cholinium (Ch^+^) and 1‐butyl‐3‐methylimidazolium (Bmim^+^) cationic additives has remarkably improved the reversibility of Zn anode in aqueous electrolytes with high CE and prolonged cycling stability.^[^ ^8^ ^]^ Meanwhile, the above‐mentioned side‐reactions (e.g., hydrogen evolution, corrosion, etc.) could also be greatly suppressed by the addition of additives in AZMBs.^[^ ^9^ ^]^ In principal, the Zn dendrite growth involves the nucleation and deposition processes, where the desolvation behavior plays a decisive role for lateral plating. The inhomogeneous nucleation of Zn deposits would elevate local electrical fields, leading to strong adsorption of Zn^2+^ and further aggregate dendrite growth. Thus, a versatile electrolyte additive should be considered to regulate the solvation structure, modulate the distribution and charge transfer of Zn ions at the interface, and generate a robust interphase to isolate the Zn anode from the reactive water molecules.

Ammonium‐based cations, known for their tunable molecular structures, have been extensively studied as redox‐inactive additives in electrolytes, especially in AZMBs. The properties of these cations can be easily adjusted by modifying the alkyl groups attached to the nitrogen atom, which allows for fine‐tuning of the physicochemical properties of the electrolytes, such as viscosity and ionic conductivity. This tunability offers a cost‐effective and sustainable strategy to optimize the performance of AZMBs. For example, a low‐cost ammonium acetate (NH_4_Ac) was first used as an additive, in which the NH_4_^+^ induces a dynamic electrostatic shielding layer around the abrupt Zn protuberance to homogenize the Zn deposition, and the Ac^−^ anion acts as an interfacial pH buffer to suppress the proton‐induced side reactions and the precipitation of insoluble by‐products.^[^ ^10^ ^]^ Yao et al. utilized the triethylmethyl ammonium cation as additive in the ZnCl_2_ and ZnSO_4_ hybrid electrolytes and achieved long‐term and highly reversible Zn plating/stripping.^[^ ^11^ ^]^ Cao et al. investigated the tetramethylammonium cations in ZnSO_4_ electrolyte, achieving lateral Zn deposition with improved Zn reversibility.^[^ ^12^ ^]^ Our recent work also demonstrated the use of methylammonium acetate as an electrolyte additive to enhance the reversibility of Zn anode, illustrating the specific role of acetate anions competitively engaging the Zn^2+^ solvation structure, reducing the water reactivity and promoting the anion‐derived interphase in aqueous electrolyte.^[^ ^13^ ^]^ Actually, the application of ammonium‐based cations as additives in electrolytes provides an effective strategy to improve the overall performance of AZMBs, with the advantages of being cost‐effective and sustainable. These additives work through mechanisms such as designing electrostatic shielding layers, water‐poor double electric layers (EDL), in situ solid electrolyte interface (SEI) layers, and regulating the solvation sheath of Zn^2+^ ions, while the impact of the specific molecular structures of these cations on improving the Zn reversibility is still not fully understood and the selection for electrolyte additive is indeed random. Especially, limited works focus on the cation chemistry and their mechanisms in regulating the interfacial chemistry of Zn anode. In this context, it is essential to establish guidelines for the screening of these cationic additives to understand the mechanism and interaction between the cationic additives and the metal electrode surface, which their identical structures are pivotal for optimizing the performance and sustainability of Zn anode in AZMBs.

Herein, the cationic chemistry of electrolyte additives has been comprehensively investigated by adjusting the spatial structure of four typical ammonium‐based cations, i.e., methylammonium (R1N^+^), dimethylammonium (R2N^+^), trimethylammonium (R3N ^+^), and tetramethylammonium (R4N ^+^), screening different localization state of positive charge. The effect of molecular structure on regulating Zn^2+^ diffusion and Zn deposition are initially pioneered to investigate, and theoretical calculations reveal that the geometry structure of the cation has a direct impact on the regulation of molecular adsorption layer on Zn anode surface. Electrochemical measurements verify that the presence of cationic adsorption layer is beneficial to regulate solvation shell structure in promoting the Zn deposition and inhibition of side reactions, thus improving the reversibility of Zn plating/stripping reactions. As a result, the Zn||Zn symmetric cell and Zn||Cu asymmetric cell delivered prolonged cycling life and improved Coulombic efficiencies. Moreover, the full cells coupled with the polyaniline (PANI), Na_3_V_2_(PO_4_)_3_, and V_2_O_5_ cathodes exhibit higher capacity retention and lower self‐discharge, displaying the practical feasibility of the optimized electrolytes employed with ammonium‐based cations. These results suggest that the cation chemistry of additives can manipulate the interfacial chemistry and improve the reversibility of Zn anode in aqueous electrolyte, offering a new avenue toward the design of advanced electrolytes.

## 2 Results and Discussion

**Figure 1:**
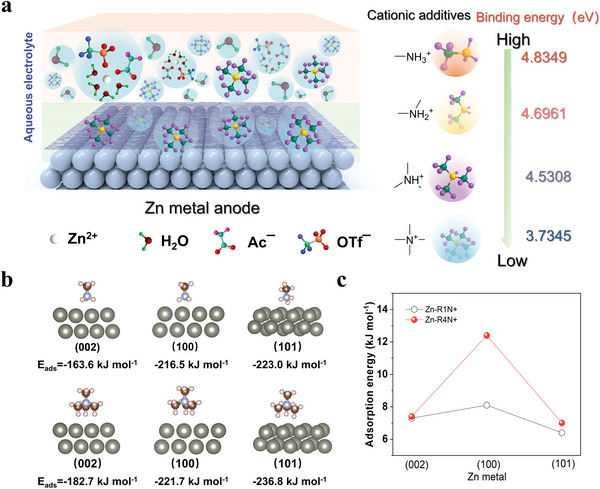
Theoretical understanding of the ammonium‐based cationic additives. a) Molecular formulas and binding energies (BE) of R1N^+^, R2N^+^, R3N^+^and R4N^+^ cations to OTf^−^ anion. b) Adsorption energy of R1N^+^ and R4N^+^ cations on the Zn (002), (100), and (101) facets. c) Zn atom adsorption energy on different crystal planes after R1N^+^ and R4N^+^ was absorbed on the Zn slab.

Although the cationic additive is widely used in improving the reversibility of Zn anode, the rational design of the molecular structure is still very challenging.^[^ ^8^, ^11^, ^14^ ^]^ The key obstruction comes from the possibility of structural modification of cations. By regulation of the structure of the ammonium‐based cations (R*_x_* N^+^), the localization state and binding energy of the related R*_x_* N^+^ and anions could be regulated. As known, larger cations with a low delocalization state of positive charge means they will have a low Gutmann acceptor number value, and the binding energy between cations and anions will be reduced. Since triflate (OTf^−^) is the main anion in the widely‐adopted zinc triflate (ZnOTf) aqueous electrolyte, the Density Functional Theory (DFT) calculations are initial to evaluate the binding energy between different R*_x_* N^+^ cations and OTf^−^. As shown in **Figure** **1**a, within different R*_x_* N^+^, their corresponding binding energies between R*_x_* N^+^ and OTf^−^ are estimated to be −4.8349, −4.6961, −4.5308, and −3.7345 eV, respectively. As the spatial size of R*_x_* N^+^ increases, the binding energies with OTf^−^ become lower.^[^ ^15^ ^]^ Accordingly, the spatial size of a quaternary ammonium (R4N^+^) is much larger than that of bare Zn^2+^, and the binding energy between R4N^+^ and OTf^−^/Ac^−^ will be lower than that of Zn^2+^, regulating the anion‐involved Zn^2+^ solvation structure. Meanwhile, the shielding effect of cations is strongly related to the localization state of positive charge in R_x_N^+^ cation, preventing the aggregation of Zn ion flux, which is beneficial for Zn atom diffusion and modulating Zn plating. This provides a great chance to manipulate the ion–solvent and ion–ion interactions, thus affecting the electric charge and adsorption behavior at the metal/electrolyte interface.^[^ ^16^ ^]^

Besides, the functions of molecular adsorption layer constructed by cationic additives on Zn electrode surface, resulting from the electrostatic shielding effect, spatially reduces the contact capability between Zn metal and water, consequently inhibiting the side reactions. The electrostatic shielding effect of the additive molecule may be affected by the localization state of positive charge. DFT calculations were also conducted to investigate the adsorption energy of cations on different facets of Zn. The results in Figure S1 (Supporting Information) clearly demonstrated that the structure of the cation has different adsorption behaviors on the Zn crystal planes. As observed, the adsorption energy of R1N^+^, R2N^+^ and R3N^+^ cations on the Zn(002), (100) and (101) planes increased when the size become larger, indicating the more significantly favorable adsorption that aid in minimizing contact between the Zn electrode and water.^[^ ^17^ ^]^ This can be attributed to the increased electrostatic polarity of the additive cation in the presence of electron‐donating methyl groups. However, the adsorption energy for R4N^+^ on the three exposed Zn surfaces demonstrated a decrease, which is only slightly higher than that of R1N^+^. Specifically, Figure 1b shows the spatial structure of the adsorbed methylammonium cations on Zn(002), (100), and (101). The structure of the adsorbed R4N^+^ is similar to that of the gas phase ion with retained tetrahedral structure.^[^ ^18^ ^]^ The other methylammonium cations adsorbed in a different way with the hydrogen closest to the surface, suggesting that the molecular structure of cationic additive can be an important factor of affecting the adsorption behaviors on Zn surface. In addition, the adsorption energy of R4N^+^ cation on the Zn (101) facet is higher than that on Zn (100) and (200). This confirms the preferential interaction of R4N^+^ cation with Zn (101) facet, providing to a more protective effect on the Zn (101) facet. Since the adsorbed cationic additive on the electrode surface will change the deposition rate of Zn^2+^, the specific adsorption energy of Zn ions on different planes in the presence of R1N^+^ and R4N^+^ adsorption was further investigated. As depicted in Figure 1c, both the R1N^+^/Zn(101) surface and R4N^+^/Zn(101) surface have an adsorption energy with Zn^2+^ lower than that on Zn(100) and (200), indicating the lowest growth rate and possibility on the (101) orientation facets. However, the R4N^+^/Zn(100) surface has a much higher adsorption energy with Zn^2+^ than that of R1N^+^/Zn(100) surface, leading to the fast growth rate on the (100) crystal plane and more exposure on other facets. As a result, the (101)‐textured Zn maintains a stable growth with fast mass transfer kinetics in the presence of R4N^+^ cationic additive. This result illustrates that not only the localization state of positive charge in the cationic additives but also the spatial occupation of the cations can regulate the Zn ions flux, and the low delocalization density of positive charge will be one of the promising one.^[^ ^19^ ^]^

**Figure 2:**
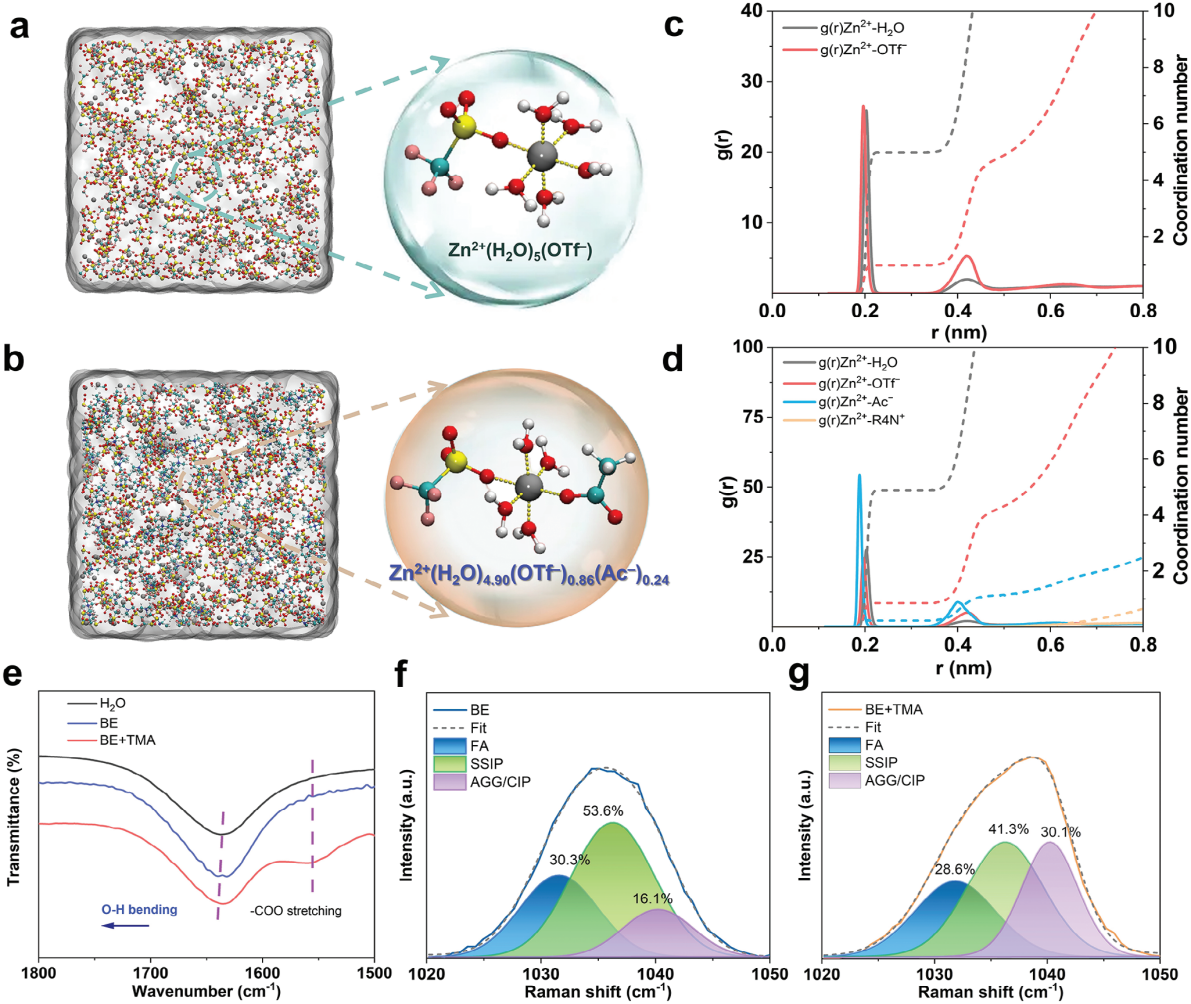
Solvation structures of the aqueous electrolytes. Simulated snapshots for a) BE and b) BE+TMA electrolytes. c, d) Radial distribution function [g(r)] and corresponding integrated coordination numbers [n(r)] obtained from MD simulations. e) FTIR spectra of pure water, BE, and BE+TMA electrolytes. f,g) Raman spectra in the wavenumber region of the SO3 stretching modes.

As screened, taking R4N^+^ as a cationic model, exemplified aqueous electrolytes of 2 m ZnOTf (m: molality) with 1 and 2 m tetramethylammonium acetate (TMA) additives, denoted as BE+TMA and BE+2TMA, respectively, were compared with the blank electrolyte (details in Experimental Section). The pH and ionic conductivity values of the electrolytes with additives were measured to evaluate their impacts on the properties of electrolytes. As shown in Figure S2 (Supporting Information), increasing the additive concentration in the electrolyte typically raises its pH, but can also reduce ionic conductivity due to potential changes in the Zn^2+^ concentration. To illustrate the solvation structure evolution of these aqueous electrolytes, molecular dynamics (MD) simulations were performed. The solvation structure in BE electrolyte contains five H_2_O molecules and one OTf^−^ anion coordinated with one Zn^2+^ cation (**Figure** **2**a). However, the addition of TMA leads to the structural rearrangement of Zn^2+^ with the participation of Ac^−^ anions (Figure 2b). Correspondingly, the radial distribution functions (RDFs) and coordination number (CN) distribution functions were analyzed, as depicted in Figure 2c,d. In the BE electrolyte, there are two coordination peaks at ≈1.96 and ≈2.03 Å, attributed to the Zn^2+^─O (H_2_O) and Zn^2+^─O (OTf^−^), respectively. In comparison, a sharp peak at ≈1.88 Å related to the Zn^2+^─O (Ac^−^) was observed, confirming that the Ac^−^ anions participated in the solvation shell with stronger interaction. According to the CN distribution results, the primary solvation cluster is calculated to be Zn^2+^(H_2_O)_5_(OTf^−^), while the Zn^2+^(H_2_O)_4.90_(OTf^−^)_0.86_(Ac^−^)_0.24_ cluster is formed in the presence of additive, modulating the solvation shell structure and suppressing the reactivity of active water in the solvation shell.^[^ ^13^ ^]^

Fourier transform infrared spectroscopy (FTIR) and Raman spectroscopy were performed to physically characterize the solvation shell structure evolutions. The FTIR spectra clearly demonstrate the ─CF_3_ and ─SO_3_ stretching vibration in two electrolytes (Figure S3, Supporting Information). Meanwhile, the characteristic peaks from the O─C═O (≈1558 and ≈1489 cm^−1^) and the C─N (≈1422 cm^−1^) stretching vibrations are observed in the modified electrolyte, implying the existence of TMA additive. Three characteristic peaks, corresponding to the “network water” (NW), “intermediate water” (IW), and “multimer water” (MW) molecules, respectively (Figure S4, Supporting Information), were observed, in which the “network water” appeared as the dominant.^[^ ^13^, ^20^ ^]^ The proportion of MW and IW located at higher frequency shows downward trend, whereas that of NW with strong H‐bond gradually increases with adding TMA additive. Meanwhile, the peak at ≈1640 cm^−1^, corresponding to the H─O bending vibration of H_2_O, experiences a slight blueshift, indicating the increased bond energy of H─O (Figure 2e). These results indicate that the additive can stabilize the water cluster via stronger H‐bond interaction and decrease the free water.^[^ ^21^ ^]^

Raman spectra of the BE and BE+TMA electrolytes (Figure S5, Supporting Information) prove the existence of TMA additive. As shown in Figure 2f,g, the ─SO_3_ stretching vibration can be spilt into three peaks related to the “free anion” (FA), “solvent‐separated ion pair” (SSIP), and “contact ion pair/aggregate” (CIP/AGG). Compared to the BE, the percentage of CIP/AGG dramatically increased from 16.1% to 30.1% in BE+TMA electrolyte, suggesting the TMA encourages the evolution of solvation shell, which is beneficial for the SEI formation derived from the reduction of anionic complexes.^[^ ^22^ ^]^ The R4N^+^ cation with a symmetric spatial structure could mitigate dendrite growth via the electrostatic shielding effect, whereas the OTf^−^ anion can induce the formation of robust solid–electrolyte interphase layers. The synergetic effect between the cation and anion might significantly improve the reversibility of Zn anode.

**Figure 3:**
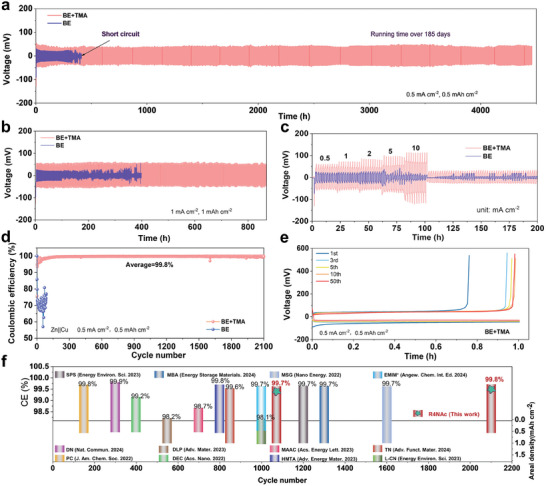
Reversibility of Zn metal anode in BE and BE+TMA electrolytes. a,b) Reversable tripping/plating tests of the Zn||Zn symmetric cells at 0.5 mA cm^−2^/0.5 mAh cm^−2^ and 4 mA cm^−2^/4 mAh cm^−2^. c) Rate performance of the Zn||Zn cells at various current densities in 1 h for each step. d) Coulombic efficiencies (CEs) of the Zn||Cu asymmetric cells at conditions of 0.5 mA cm^−2^/0.5 mAh cm^−2^. e) Selected voltage profiles during the cycling. f) Comparison of published Zn plating/stripping CE results upon cycling on Cu substrates in electrolytes with different additives (detailed information can be referred to the supplementary information).

The electrochemical performance of Zn anode was investigated to evaluate the reversibility of Zn plating/stripping reactions in the electrolytes with/without additive. Initially, Zn||Zn symmetric cells were tested in the BE, BE+TMA, and BE+2TMA electrolytes at 5 mA cm^−2^ under 5 mAh cm^−2^, respectively. As shown in Figure S6 (Supporting Information), the BE electrolyte led to a short circuit within 10 h, while the BE+2TMA electrolyte not only showed the highest polarization voltage but also poor cycle stability with noticeable voltage irregularities occurring within 80 h. In contrast, the BE+TMA electrolyte demonstrated stable cycling life for over 135 h, indicating that the BE+TMA electrolyte is the superior concentration to enhance the reversibility of Zn anode. **Figure** **3**a presents the long‐term cycling performance of Zn||Zn symmetric cells using BE and BE+TMA electrolytes at 0.5 mA cm^−2^. As observed, the cell with BE only cycled for 400 h (200 stripping/plating cycles) due to the short circuit, while the cell with TMA additive exhibited a steady voltage profile without any dendrite, reaching a prolonged lifespan >4460 h (over 185 days), which is several times longer than that of the methylammonium acetate additive.^[^ ^13^ ^]^ Elevating the current density to 1 mA cm^−2^ under 1 mAh cm^−2^ (Figure 3b), the cell can still be stabilized over 870 h without short circuit, which is superior to that with BE (100 h). At a higher current density of 5 mA cm^−2^ and areal capacity of 5 mAh cm^−2^, the Zn||Zn cell in BE+TMA can cycle steadily for 135 h, much longer than that of BE (only 10 h) (Figure S7a, Supporting Information). Even increasing the areal capacity to 20 mAh cm^−2^, the Zn||BE+TMA||Zn cell still kept working for 280 h. In sharp contrast, the Zn||Zn symmetric cell in BE cannot survive for two cycles, proving the presence of TMA additive significantly improves the reversibility of Zn anode (Figure S7b, Supporting Information). Furthermore, the rate performance of Zn||Zn cells in electrolytes with/without TMA was also studied at various current densities ranging from 0.5 to 10 mA cm^−2^ (Figure 3c). Clearly, the cell employing with the BE+TMA electrolyte delivers a steadier voltage profile than that of baseline electrolyte. When the current density was increased to 2 mA cm^−2^, the cell using BE electrolyte immediately fails.

The asymmetric cells of Zn||Cu were also assembled to assess the CE. At 0.5 mA cm^−2^ (Figure 3d), the Zn||Cu cell in BE+TMA electrolyte demonstrated a significantly prolonged cycling life over 2100 cycles with an extremely high average CE of ≈99.8%. However, the cell in BE exhibits rather poor CEs <70% upon the limited cycle life of 40 cycles. The voltage profiles verify the improvement in reversibility of Zn stripping and plating reactions, without obvious overpotential polarization during the initial 50 cycles in the modified electrolyte (Figure 3e). At higher current densities or specific capacities (Figure S8, Supporting Information), the cells still exhibited superior electrochemical stability with high CEs, further illustrating the additive can effectively improve the reversibility of Zn anode. Figure 3f presents a compilation of published CE data for Zn stripping/plating reaction on Cu substrates in electrolytes with various additives, and comprehensive details are provided in Table S1 (Supporting Information). The CEs are assessed based on the total Zn capacity plated and the number of cycles completed, which are key indicators of battery performance. The results underscore the superiority of ammonium‐based additives in refining the interface of Zn metal, enhancing the longevity and reversibility of Zn electrode. Remarkably, the cells tested with the TMA additive demonstrate superior stability over an extended cycle life, surpassing numerous studies in the field, which significantly boosts the durability and reversibility of the zinc electrode.

The cycled Zn electrodes were characterized to investigate the effects of TMA additive on deposition behaviors and by‐product formation. To verify the effect of cationic additive on the deposition of Zn metal, current‐dependent X‐ray diffraction (XRD) characterization was conducted in BE+TMA electrolyte with limited capacity of 5 mAh cm^−2^. The XRD profiles of the plated Zn electrodes (Figure S9, Supporting Information) show that the commercial Zn foil is highly (002) textured, which is thermodynamically reactive with aqueous electrolyte.^[^ ^23^ ^]^ With the increase of current density, the peak intensity ratio of I_(002)_/I_(101)_ is greatly reduced from 1.80 to 0.59, indicating the preferable growth of (101) textured Zn deposits. Such textured (101) Zn deposits would be beneficial for suppressing the hydrogen evolution and side reactions, reaching high reversibility of Zn anode.^[^ ^19b^ ^]^

**Figure 4:**
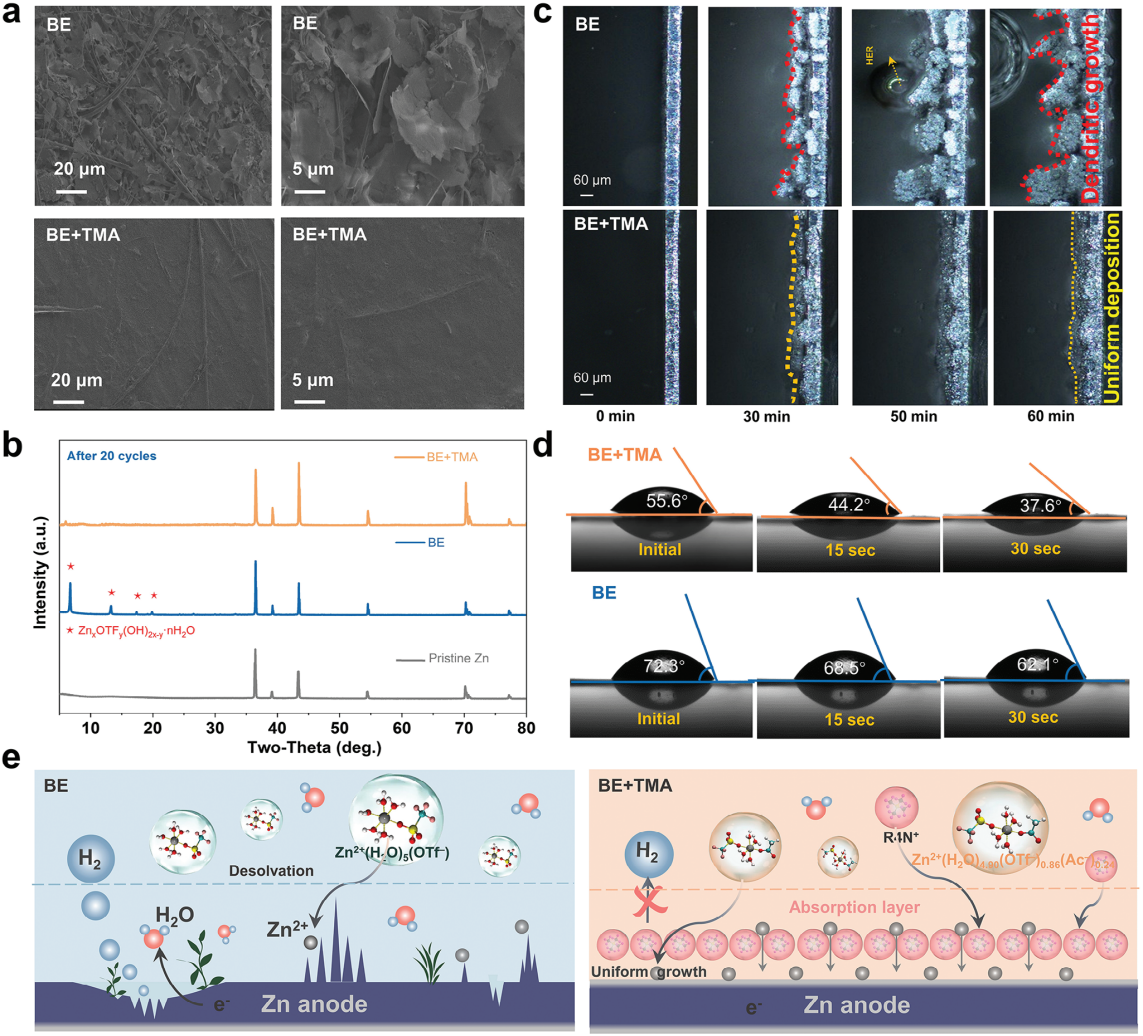
Structural evolution of Zn anodes in the different electrolytes. a) SEM images and b) XRD patterns of the cycled Zn anodes retrieved from the Zn||Zn cells. c) In situ optical microscopy images of the cross‐sectional Zn deposition morphology evolution on the Zn foil with plating times at a current density of 10 mA cm^−2^. d) Contact angle measurements of electrolytes on Zn metal foils. e) The schematic illustration of Zn deposition behaviors in BE and BE+TMA electrolytes.

Scanning electron microscopy (SEM) images were recorded on the Zn surface after plating, as presented in **Figure** **4**a. The Zn surface cycled in the BE electrolyte shows an uneven and porous surface with randomly distributed sheet‐like structure. In contrast, a smooth and dense morphology was observed on the Zn electrode when plated in BE+TMA electrolyte, indicating the homogenous deposition and growth of Zn. The XRD patterns were collected on the cycled Zn anodes in the specific electrolyte to distinguish the deposits. In Figure 4b, the Zn anode after cycling in BE+TMA displays the typical diffraction peaks as the pristine Zn without any impurity peaks detected, while distinctive characteristic peaks related to Zn*_x_* (CF_3_SO_3_)*_y_* (OH)_2_ *_x_*_‐_ *_y_* ·*n*H_2_O were observed on Zn cycled in BE, which is the main byproduct from the inevitable hydrogen evolution and corrosion reactions. Moreover, to directly verify the inhibition effect of additive on corrosion reactions, self‐corrosion test was performed by soaking the Zn electrodes in the electrolytes for 10 days. The XRD patterns were recorded on the pre‐treated electrodes, as presented in Figure S10 (Supporting Information). Clearly, due to the thermodynamic instability of Zn metal in aqueous solution, self‐corrosion reaction occurs spontaneously in contact with the aqueous electrolytes, leading to the loss of metallic Zn and the formation of irregular flakes as corrosion by‐products on the Zn surface.^[^ ^24^ ^]^ However, the Zn electrode in the optimal electrolyte with the additive also demonstrates no distinctive by‐products on the surface, proving the good anti‐corrosion ability of Zn metal. The corrosion resistance of the Zn anode in the electrolytes was evaluated by Tafel plots, as shown in Figure S11 (Supporting Information). Clearly, the Zn anode in BE+TMA presents a lower corrosion current density of 0.091 mA cm^−2^ than that in BE (0.139 mA cm^−2^), indicating the stronger corrosion tolerance of Zn metal in BE+TMA electrolyte. In the chronoamperometry (CA) curves (Figure S12, Supporting Information), under a fixed overpotential of −150 mV, the current of the Zn electrode in the BE+TMA electrolyte is smaller than that of the BE and a stable and constant 3D diffusion is achieved, resulting from the restrained diffusion of Zn^2+^ and thus harnessing the dendrite growth.

Additionally, in situ optical microscopy experiments were conducted to visually monitor the Zn deposition behaviors (Figure 4c). The digital photographs of the cross‐sectional Zn electrode in BE+TMA electrolyte demonstrate a uniform and dense Zn deposition process at 10 mA cm^−2^ for 60 min. In BE electrolyte, obvious Zn protrusions emerge on the Zn surface after 30 min plating and continuously gathers in the later 50 min. These protuberances will finally develop into dendrites and pierce through the separator, inducing to short circuit. At the same time, bubbles can be clearly seen on the Zn surface at 50 min and become larger, illustrating the occurrence of hydrogen evolution reaction in BE and leading to low CE and by‐products formation. Furthermore, the dynamic contact angle was measured to determine the zincophility of BE and BE+TMA electrolytes on the Zn surface (Figure 4d). The BE+TMA electrolyte exhibits lower contact angle at initial state, and decreases to 37.6° after 30 s, while the BE electrolyte has higher contact angles, suggesting that the wettability of electrolyte to the Zn anode is improved with the additive. The superior wettability of BE+TMA on the Zn surface reduces the interfacial free energy between the Zn metal and the aqueous electrolyte, leading to the formation of a uniform plating surface.

The nature of substrates has a critical impact on the reversibility of Zn deposition. Additional titanium (Ti) foil and porous carbon cloth (CC) were investigated as working electrodes for Zn deposition. As displayed in Figure S13 (Supporting Information), the Zn||Ti asymmetric cell using the BE+TMA demonstrated higher CEs than that in BE, which can be cycled for >400 h at 0.5 mA cm^−2^. Moreover, the Zn||CC cell also possessed longer cycling life with high CEs in the optimized TMA‐based electrolyte. The SEM images recorded on the Zn deposited Ti and CC substrates show the uniform deposition of Zn metal on substrates, without the formation of flake‐like by‐products. These results strongly validate the versatile application of the electrolytes for highly reversible Zn anode. To further confirm the versatility of cationic strategy on the design of advanced aqueous electrolyte for AZMBs, an electrolyte containing 2 m ZnSO_4_ and 1 m TMA (denoted as ZnSO_4_+TMA) was also prepared, as shown in Figure S14 (Supporting Information). In the ZnSO_4_+TMA, the Zn||Zn cell can be cycled over 1600 h at 0.5 mA cm^−2^, while the cell only survived in 330 h in ZnSO_4_ electrolyte. At higher current densities and capacities, the cells in the optimized electrolyte still demonstrated remarkably improved stability. Therefore, the incorporation of ammonium‐based cationic additives forms an adsorptive interphase that significantly reduces the tip effect on Zn surfaces, ensuring uniform Zn^2+^ adsorption on the entire electrode. Consequently, with the TMA‐containing electrolyte, metallic Zn is uniformly plated without dendrite growth, and side reactions such as hydrogen evolution and corrosion are effectively minimized, as schematically depicted in Figure 4e. In principle, the Zn electrode, benefiting from a low nucleation overpotential and a uniformly distributed electric field, neutralizes the adverse effects of Zn dendrite formation and other parasitic reactions during deposition.^[^ ^25^ ^]^ This leads to the facilitation of highly reversible Zn plating/stripping reactions, achieving high CEs.

**Figure 5:**
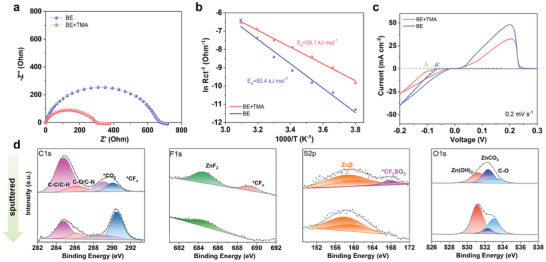
Interfacial behaviors of Zn anode. a) Electrochemical impedance spectroscopy of Zn||Zn cells recorded at fresh state. b) The de‐solvation activation energies of Zn anode in the electrolytes calculated by the Arrhenius equation. c) CV curves of recorded at 0.2 mV s^−1^. d) The XPS depth profiles for C1s, F1s, S2p, and O1s of Zn anode surface after cycling.

The interfacial chemistry in regulating the Zn deposition process was also investigated. **Figure** **5**a displays the Nyquist plots of the Zn||Zn symmetric cells in BE and BE+TMA electrolytes at initial state. The charge transfer resistance (R_ct_) in modified electrolyte was significantly reduced from 682 to 315 Ω compared to that of BE. It is generally believed that the desolvation process of hydrated Zn^2+^ is regarded as the rate‐limiting step for the charge transfer at electrode surface. The regulation of adsorption layer on the electrode surface will also enhance the desolvation process. Accordingly, the desolvation energies of the hydrated Zn^2+^ in these electrolytes were evaluated. Since the contributions of liquid‐phase transport in the aqueous electrolyte and solid‐phase transport at the electrode surface are neglectable, the desolvation of hydrated Zn^2+^ to naked Zn^2+^ is the dominant energy barrier in the charge transfer process during Zn plating. Thus, by measuring the R_ct_ values at various temperatures, the activation energy (E_a_) can be calculated through the Arrhenius equation (see details in the Experimental part), as shown in Figure 5b. The E_a_ for the Zn electrode in BE+TMA electrolyte is calculated to be 38.1 kJ mol^−1^, which is smaller than that in BE electrolyte (55.4 kJ mol^−1^), indicating the lower desolvation energy barrier and faster desolvation process of the hydrated Zn^2+^ at electrode interface, resulting in the reduced corrosion current, thus suppressing corrosion. Figure 5c shows the cyclic voltammetry (CV) curves of Zn||Cu asymmetric cells in different electrolytes. The Zn nucleation overpotential for the TMA‐containing electrolyte was higher than that of the BE electrolyte, suggesting the additive can lead to smaller nucleation radius and more homogenous deposition. The chemical composition of the SEI on cycled Zn anode was characterized by high‐resolution X‐ray photoelectron spectroscopy (XPS) before and after Ar^+^ sputtering. As seen in Figure 5d, the C1s spectra on the surface of cycled Zn can be deconvoluted into four characteristic peaks related to the C─C/C─H, C─O/C─N, O─C═O, and CF_x_ species at ≈284.8, ≈286.2, ≈288.6, and ≈290.1 eV, respectively. After etching, the intensity of the peak related to the CF_x_ species was relatively increased, due to the incomplete decomposition of OTf^−^ anions at the surface. The F1s spectra indicate the presence of organic CF_x_ species (≈689.2 eV) and inorganic ZnF_2_ (≈684.4 eV). Only signals belonging to ZnF_2_ (≈685.0 eV) and ZnS (≈162.2 eV) species were detected from the high‐resolution F1s and S2p spectra, confirming the reduction of anions for the SEI formation. The O1s spectra demonstrate the presence of trace amounts of ZnCO_3_ and Zn(OH)_2_ inorganic species. The N species related to the cation's decomposition are rarely detected on the electrode before and after etching (Figure S15, Supporting Information), suggesting its rare involvement in SEI formation.^[^ ^13^ ^]^ However, the spectra shows much reduced intensity in C1s, F1s and S2p peaks on both the fresh and sputtered samples, indicating the thickness of the interphase layer formed in BE+TMA electrolyte is relatively thin, with respect to the methylammonium acetate additive.^[^ ^13^ ^]^

**Figure 6:**
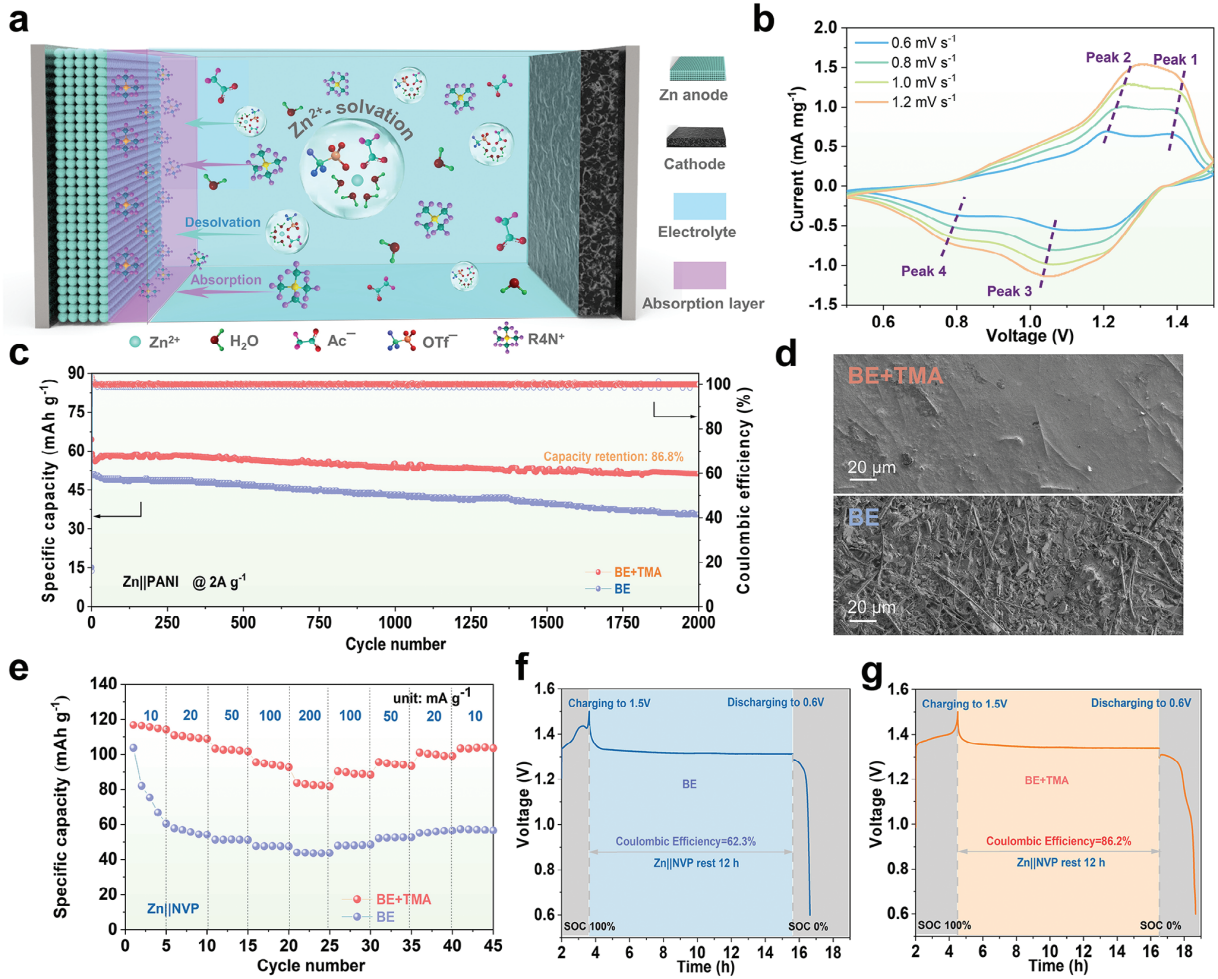
Electrochemical performance of full cells. a) Schematic illustration of Zn||PANI cell in BE+TMA electrolyte. b) CV curves of Zn||PANI cell in BE+TMA electrolyte at various scan rates. c) Long‐term cycling stability at 2 A g^−1^. d) SEM images recorded on Zn metal anode depicted from cycled Zn//PANI cells. e) Rate performance of Zn//NVP cells. f,g) The self‐discharge behaviors of Zn//NVP cells, showing the self‐corrosion effect.

To practically versify the feasibility of the modulation strategy in aqueous zinc full cells, BE and BE+TMA electrolytes were first tested in Zn||polyaniline (PANI) full cell, as depicted in **Figure** **6**a. The PANI nanofiber structure were characterized by XRD and SEM analysis (Figure S16, Supporting Information). The CV curves of the Zn||PANI full cell in BE+TMA were recorded at various scan rates (Figure 6b). Two pairs of redox peaks can be clearly observed, and exhibit slight polarization when increasing the scan rate, indicating the typical redox reaction and fast kinetic of PANI cathode.^[^ ^26^ ^]^ Figure 6c shows the cycling performance of the full cells at 2.0 A g^−1^ in BE and BE+TMA, in which the cell in BE+TMA demonstrated higher cycling stability, with a capacity retention of 86.8% over 2000 cycles and a high average CE (>99.9%). However, the cell in BE only had a capacity retention of 63.7% with violent fluctuations in CEs. The selected voltage profiles during the cycling are presented in Figure S17 (Supporting Information), suggesting the superior reversibility of the cell in BE+TMA. The SEM images depicted in Figure 6d showcase the Zn anode with the uniform and reversible deposition behavior of Zn anode in the modified electrolyte. When increasing the mass loading of PANI in cathode to 4.5 mg cm^−2^, the cell can still have a capacity retention of 81.6% after 2000 cycles (Figure S18, Supporting Information). Notably, the choice of carbon cloth as current collector can lead to improved reversible capacity compared to stainless steel foil owing to the high tightness between active materials and current collector.^[^ ^27^ ^]^ Considering the fact of slightly increased polarization voltage during Zn stripping/plating reactions in TMA‐modified electrolyte, the energy efficiencies of full cells were determined at different cycles, as displayed in Figure S19 (Supporting Information). It is seen that the incorporation of TMA additive into the electrolyte has negligible impact on the energy efficiency of Zn||PANI cells at initial 100 cycles. However, the efficiency is significantly higher in the BE+TMA compared to the BE, highlighting the advantage of this additive for AZMBs that are both long‐lasting and highly efficient.

To further prove the versatility of this electrolyte in aqueous Zn full cells, Zn||Na_3_V_2_(PO_4_)_3_ (NVP) cells were also fabricated and tested employing BE and BE+TMA (Figure S20, Supporting Information; Figure 6e). The Zn||NVP cells in BE+TMA demonstrate a significantly improved cycling stability at 50 mA g^−1^ and robust rate performance up to 200 mA g^−1^ with typical charging/discharging plateaus of NVP cathodes for aqueous Zn storage. It is worth noting that the Zn||NVP cell exhibits higher capacity in BE+TMA electrolyte than that of BE electrolyte, which can be attributed to the well‐suppressed structure degradation and dissolution of NVP materials, leading to higher reversible capacity during the (de)intercalation process of Zn^2+^. Meanwhile, an intrinsic challenge of persistent self‐discharge has risen as a frequently overlooked issue in vanadium‐based AZMBs.^[^ ^28^ ^]^ As shown in Figure 6f,g, the Zn||NVP full cell delivered a capacity retention of 86.2% when charged to 1.5 V at a low current density (50 mA g^−1^) after 12 h rest, higher than that of BE (62.3%). Adopting more practical cathode of V_2_O_5_, the Zn||V_2_O_5_ full cells were compared in the BE and BE+TMA electrolytes (Figure S21, Supporting Information). At a high current density of 2 A g^−1^, the modified electrolyte can significantly improve the cycle stability and efficiency of full cells under higher mass loading. These results clearly reveal that the strategy of cationic additives in aqueous electrolyte design can practically improve the reversibility of Zn metal anode, consequently approaching improved battery efficiency and stability.

## 3 Conclusion

In summary, an in‐depth analysis of the role of interfacial dynamics and adsorption layers through an ammonium‐based cationic additive strategy in enhancing zinc anodes has been proposed and investigated. The results uncover that the molecular architecture of cationic additives, particularly the distribution of positive charge, significantly influences their adsorption characteristics and interaction with zinc metal, thereby facilitating the oriented growth of zinc crystal planes, as fully confirmed by theoretical simulations. The symmetric spatial configuration of the quaternary methylammonium cationic additive (R4N^+^) confers an ultralong cycle life of over 4400 h at 0.5 mA cm^−2^ and a high average CE of 99.8% at a current density of 0.5 mA cm^−2^, stabilizing for 2100 cycles. The marked enhancement in zinc reversibility is credited to the suppression of interfacial corrosion and the dendrite‐free Zn deposition. Leveraging these advancements, full cells coupled with various cathodes in the additive‐optimized electrolyte exhibit enhanced long‐term cycling stability. This work provides novel insights into the zinc anodes through molecular engineering with cationic additives in aqueous electrolytes for high‐performance zinc batteries.

## Conflict of Interest

The authors declare no conflict of interest.

## Supporting information



Supporting Information

## Data Availability

The data that support the findings of this study are available from the corresponding author upon reasonable request.
