# Supplementary material for: Screening Ammonium‐Based Cationic Additives to Regulate Interfacial Chemistry for Aqueous Ultra‐Stable Zn Metal Anode
Source: Adv Sci (Weinh). 2024 Sep 28;11(43):2407102. doi: 10.1002/advs.202407102 (PMC11578347; doi:10.1002/advs.202407102)
Supplement: Supplementary file 1 — Supporting Information [file ADVS-11-2407102-s001.docx]

Supporting Information

**Screening Ammonium-Based Cationic Additives to Regulate Interfacial Chemistry for Aqueous Ultra-Stable Zn Metal Anode**

*Leilei Zheng, Huihua Li,* Mingbo Gao, Keer Huang, Jian Wang,* Long Su, Lei Li, Hongzhen Lin, Xinpei Gao, Zhengqing Liu, and Huang Zhang**

L. Zheng, Prof. H. Li, M. Gao, Prof. H. Zhang

Key Laboratory of Engineering Dielectric and Applications (Ministry of Education), School of Electrical and Electronic Engineering, Harbin University of Science and Technology, Harbin 150080, P. R. China

E-mail: li.huihua@hrbust.edu.cn; zhang.huang@hrbust.edu.cn

L. Zheng, K. Huang, Prof. L. Li, Prof. Z. Liu, Prof. H. Zhang

Institute of Flexible Electronics, Northwestern Polytechnical University, Xi'an 710072, P. R. China

Dr. J. Wang, Prof. H. Lin

*i*-lab, & CAS Key Laboratory of Nanophotonic Materials and Devices, Suzhou Institute of Nano-Tech and Nano-Bionics, Chinese Academy of Sciences, Suzhou 215123, Jiangsu, China

E-mail: wangjian2014@sinano.ac.cn

Dr. J. Wang

Helmholtz Institute Ulm (HIU), Ulm D-89081, Germany

Karlsruhe Institute of Technology (KIT), Karlsruhe D-76021, Germany

E-mail: jian.wang@kit.edu

Dr. L. Su

Key Laboratory of Colloid and Interface Chemistry (Ministry of Education), Shandong University, Jinan, P. R. China

Prof. X. Gao

Key Laboratory of Advanced Materials in Tropical Island Resources (Ministry of Education), School of Chemical Engineering and Technology, Hainan University, Haikou, P. R. China

**Experimental Section**

***Material Synthesis***

The Na_3_V_2_(PO_4_)_3_ material (NVP) was synthesized by solid-state method.^S[1]^ Typically, 2 mmol NH_4_VO_3_ (99%, Sigma-Aldrich), 3 mmol CH_3_COONa·3H_2_O (99%, VWR), and 4 mmol NH_4_H_2_PO_4_ (99%, Alfa) were dissolved in 50 mL 2 wt.% citric acid (C_6_H_8_O_7_·H_2_O, 99%, Alfa) aqueous solution and then dried at 80°C overnight. The precursors were grounded and pre-heated at 350 °C for 6 h, then calcined at 800 °C for 12 h under a steady Ar flow. The NVP black powder was eventually collected.

The polyaniline (PANI) material was synthesized according to the literature.^S[2]^ Specifically, 0.365 mL of aniline (C_6_H_5_NH_2_ , 99%, Innochem) was added into 15 mL HCl solution (1.0 M, Innochem), and the mixed solution was stirred in an ice bath conditions for 30 minutes (below 0°C). Then, 0.228 g ammonium persulfate ((NH_4_)_2_S_2_O_8_, 99.99%, Aladdin) was firstly dissolved in 5 mL HCl (1.0 M) solution and then added into the previous mixed solution in the ice bath under stirring for 2 hours. The solution eventually turned dark green. Finally, the suspension was centrifuged and the collected solid was washed three times with deionized water and ethanol (volume ratio of 1:1). The material was dried overnight at 60 °C and ground to obtain the final PANI products.

***Electrolyte Preparation***

The baseline electrolyte was prepared by dissolving 2 mol kg^-1^ Zn(CF_3_SO_3_)_2_ (ZnOTf, 99.5%, Adamas) in Milli-Q water and denoted as blank electrolyte (BE). The optimal electrolyte was prepared by dissolving additional 1 mol kg^-1^ tetramethylammonium acetate (TMA, 98%, Aladdin) into the blank electrolyte (denoted as BE+TMA). Additionally, the electrolyte with 2 mol kg^-1^ TMA additive was also prepared (denoted as BE+2TMA). The ZnSO_4_-based electrolytes were prepared by dissolving ZnSO_4_ (99.5%, Aladdin) and TMA in same molality as BE and BE+TMA electrolytes, and denoted as ZnSO_4_ and ZnSO_4_+TMA, respectively. All the electrolytes were constantly bubbled with N_2_ for 30 minutes to remove dissolved O_2_ before use.

***Material Characterizations***

The crystal structure was characterized on a Bruker D8 X-ray Diffractometer (XRD, Bruker, D8 Advance) using Cu Kα radiation (λ = 0.154 nm). The morphology was identified by field-emission scanning electron microscopy (FE-SEM, ZEISS, Gemini SEM 300). Transmission electron microscopy (TEM, HITACHI, HT7800) was used to characterize the microstructure of PANI. X-ray photoelectron spectroscopy (XPS, PHI 5000 Versa Probe III) was used to collect elemental information on the electrode surface before and after etching at a depth of 10 nm. The binding energy was calibrated using a C1s peak (284.8eV). The electrolytes were characterized by Fourier transform infrared spectroscopy (FTIR, Bruker Tensor II), Raman spectroscopy (Bruker Vertex70) with a laser wavelength of 1064 nm. All the collected spectra were analyzed using PEAK FIT software (v4.12, Sea Solve Software. Inc.). The contact angles of the electrolytes on Zn surface were measured by Kruss DSA100 with 2 μL electrolyte for each test. The *in situ* observation of the Zn deposition on Zn electrode was performed on optical microscope (Nikon SMZ1270) at a current density of 10 mA cm^-2^.

***Electrochemical Characterizations***

The cathodes were fabricated by using PANI, NVP or commercial V_2_O_5_ powder (98%, Alfa Aesar) as active materials, and carbon black (Super C65, IMERYS) as conductive agent and polyvinylidene difluoride (PVDF, Solef 6020, Solvay) as binder (mass ratio of 8:1:1) in N-methyl-2-pyrrolidone (NMP, anhydrous, 99.5%, Sigma-Aldrich), respectively, and coated on stainless steel foil (type-316, thickness: 0.02 mm, Ø 12 mm). The electrodes were dried in air at 80 ℃ and further vacuum-dried overnight at 120 ℃. The typical mass loading of the active materials (PANI or NVP) was calculated to be 1.5~2 mg cm^-2^ unless specifically indicated. All the electrochemical tests were conducted in CR2032 coin-type cells at room temperature (~20°C). For high mass loading electrodes, carbon cloth was used as current collector due to its high conductivity and porous structure, which provide both mechanical strength and the capacity to accommodate larger amounts of active material.

Chronoamperometry (CA) and cyclic voltammetry (CV) measurements were conducted on an electrochemical workstation (CHI 660E). For Zn||Cu asymmetric cells, the CV curves were collected in the voltage range of -0.2 V~1 V at a scanning rate of 0.2 mV s^-1^. For Zn||PANI full cells, the CV curves were recorded in the voltage range of 0.5 V~1.5 V at various scanning rates from 0.6 to 1.2 mV s^-1^. CA tests were performed in Zn||Zn symmetrical cells, with the bias voltage of -150 mV, and recording time of 300 s. The electrochemical impedance spectroscopy (EIS) results were recorded on the fresh cells and cycled cells at discharged state, with a signal amplitude of 5 mV in the frequency range from 10^5^ Hz to 10^-2^ Hz.

Galvanostatic charge-discharge (GCD) tests of full cells and stripping-plating tests of Zn metal electrodes were carried out on a battery testing system (NEWARE, CT-4008T) at various current densities. Zn||Zn symmetric cells were used for ionic conductivity measurement based on the recorded EIS spectra at room temperature, operating in the frequency range of 10^-2^ Hz~10^5^ Hz. The Nyquist curve intercept provides a measure of the electrode resistance, and the ion conductivity can be calculated using the following formula:

where *σ* (S cm^-1^) is the ionic conductivity, *d* (cm) is the direct distance between two electrodes, *R_s_* is the electrolyte resistance, and *S* (cm^2^) is the electrode area.

The activation energy (Ea) of an electrochemical reaction can be determined by measuring the temperature dependence of the charge transfer resistance (Rct) derived from EIS spectra. The R_ct_ was obtained by fitting Nyquist curve with Zview software. The activation energy can be calculated by the Arrhenius equation:

where *R_ct_* is the charge transfer resistance, *A* is the frequency factor, *T* is the absolute temperature, and *R* is the molar gas constant.

Self-discharge tests were performed on the electrochemical workstation (CHI 660E) in Zn||NVP full cells. The as-fabricated cells were r charged to 1.5 V at 50 mA g^-1^ and discharged to 0.6 V after rested for 12 h. The coulomb efficiencies were calculated. Higher coulombic efficiency correlates with lower self-discharge rates in full cells.

***Molecular Dynamics Simulation***

Classical molecular dynamics (MD) simulations were conducted for various electrolytes using the GROMACS 2020.6 software and modeled adopting the General Amber force fields (GAFF) parameters to investigate the microstructures and dynamics properties of electrolytes. The geometries and charge distribution of the OTf^−^, R*_x_*N^+^ and AC^−^ were computed by the B3LYP functional of DFT calculations with 6-311++G** basis set method using the Gaussian16 program. Then, the restrained electrostatic potential (RESP) charges ^S[3]^ were fitted by antechamber tool ^S[4]^ for MD simulations. To approximate the effect of charge transfer and polarizability in the bulk phase, the partial charges of all ions were scaled by a constant of 0.75 ^S[5]^. The water molecules were described using the simple point charge extended (SPC/E) model ^S[6]^. Initial configurations with periodic boundary conditions in XYZ directions were constructed using Packmol code ^S[7]^. An energy minimization was performed to minimize the energy of the initial configuration using steepest descent method ^S[8]^ employing a convergence criterion of 500 kJ·mol^−1^·nm^−1^. NPT ensemble was performed for 30 ns to obtain the correct density of the electrolytes using the Berendsen barostat ^S[9]^. The V-rescale temperature coupling method ^S[10]^ was applied to couple the temperatures. Afterward, a 50 ns production simulation was performed in NVT ensemble for subsequent analysis. Furthermore, the particle mesh Ewald (PME) method ^S[11]^ and Lennard-Jones potential were utilized to describe long-range electrostatic interactions and van der Waals interaction with a cutoff of 1.2 nm. The LINCS algorithm ^S[12]^ was used to constrain the bond length to the equilibrium values.

***DFT Calculations***

The geometries of these molecules were all optimized with dispersion corrected density functional theory (DFT-D3) at the PBE0-D3/TZVP ^S[13]^ level using Gaussian program. In addition to the explicit water coordinated to cations, the SMD (Solvation Model Based on Density) ^S[14]^ implicit solvent model was used in all calculations to describe the solvation effect of water further. The vibrational frequency analyses were carried out for these optimized structures with the same calculation method. Density functional theory (DFT) as implemented in Vienna Ab-Initio Simulation Package (VASP) ^S[15]^ was used to optimize crystal parameters of zinc. The Perdew-Burke-Ernzerhof (PBE) ^S[16]^ exchange correlation functional was employed in these calculations. These calculations were started with a cell parameters and geometry optimization of five layers of zinc slab model structure. For structural optimization, a kinetic energy cutoff of 450 eV was used, the Brillouin zone integration was sampled with 3x3x1 Gamma K point mesh grid. The convergence threshold for atomic force during structural optimization was 0.01eV/Å. The adsorbed structure and corresponding adsorb energy of water, OTf^−^ and Ac^−^ species on the Zn slab were optimized with the same parameters as above. In order to describe the solid phase properties, the cell parameters and the three bottom layers were fixed and only the atomic positions of two top layers were optimized in these calculations. The adsorption energies (Eads) were calculated by using the following equation:

Eads = E(adsorbate/Zn) – E(adsorbate) – E(Zn)

where E(Zn), E(adsorbate), and E(adsorbate/Zn) represent the total energies for the zinc, adsorbate, and complex structure, respectively.


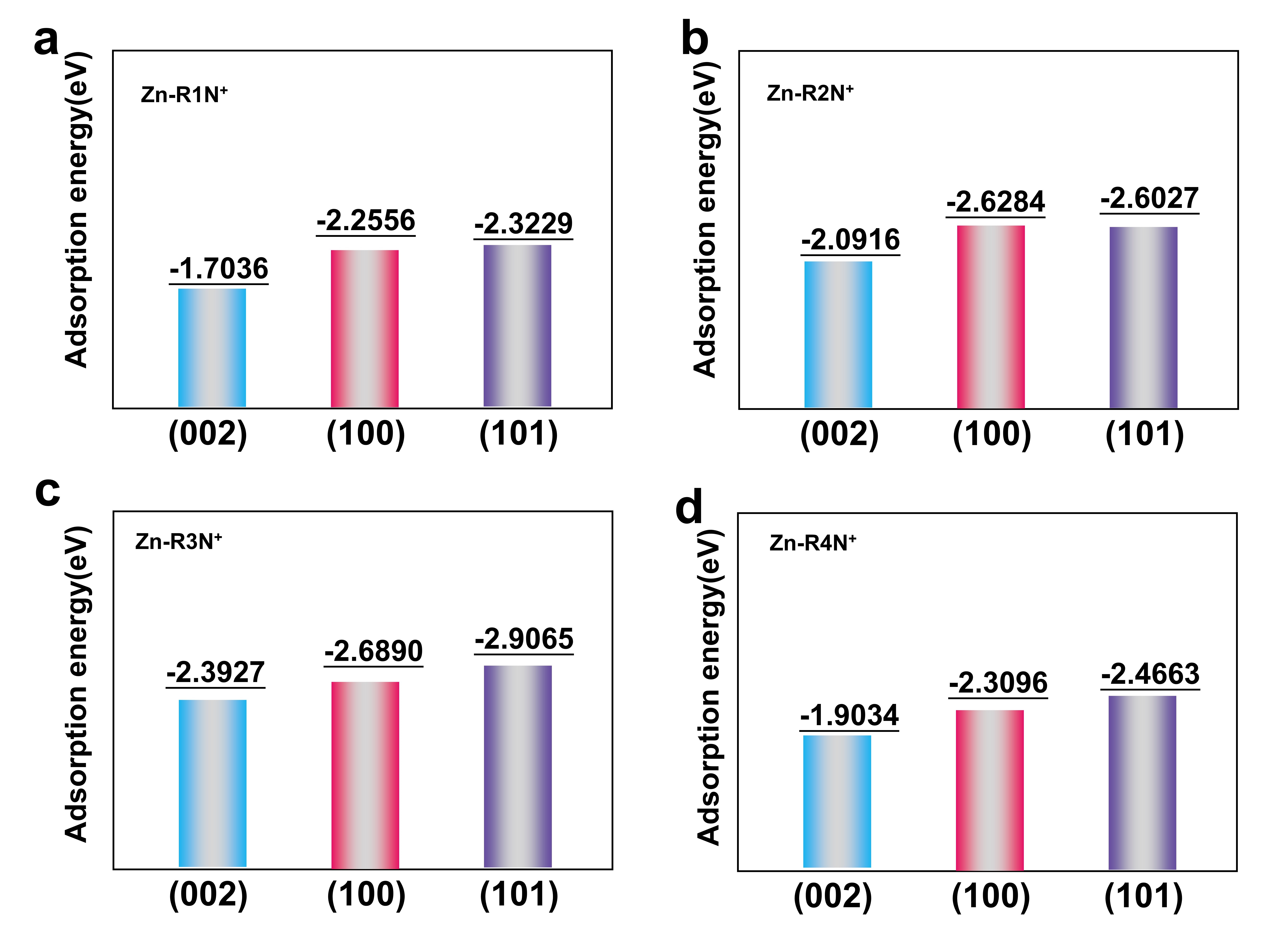


Figure S1. Adsorption energy of R1N^+^, R2N^+^, R3N^+^ and R4N^+^ cations on the Zn (002), (100), and (101) surfaces.


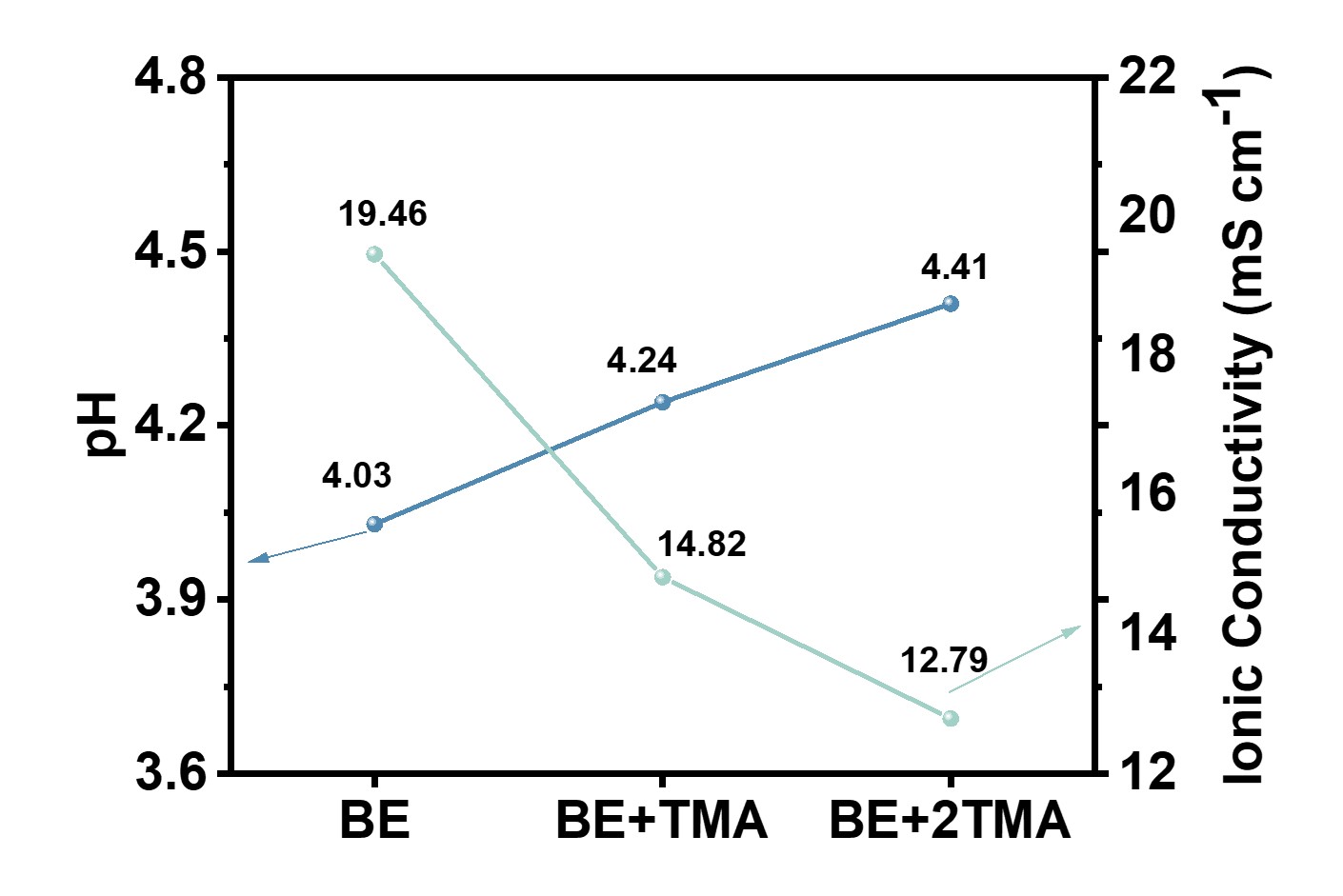


Figure S2. The pH and ionic conductivity values of BE, BE+TMA and BE+2TMA electrolytes at room temperature.


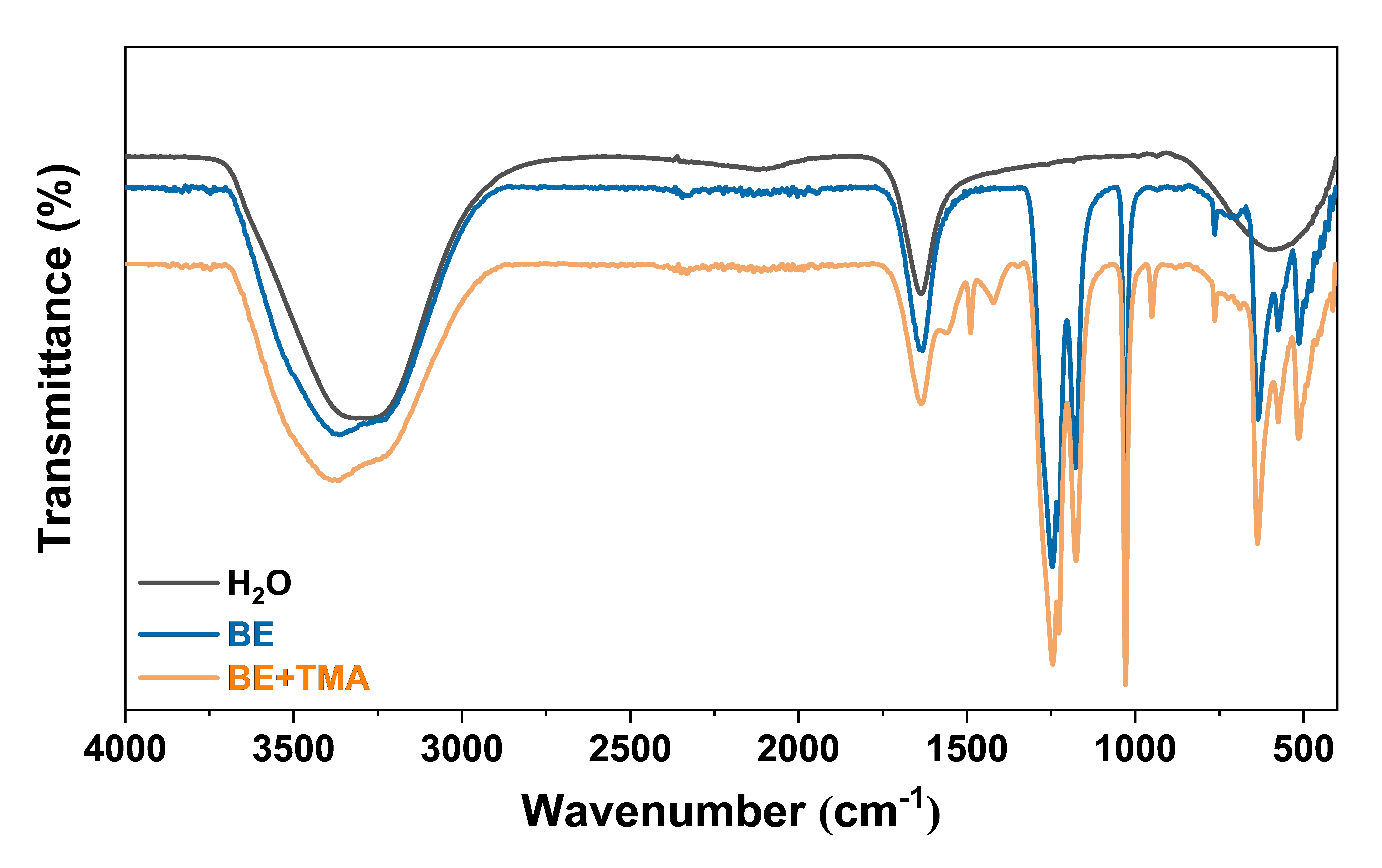


Figure S3. FTIR spectra of H_2_O, BE, and BE+TMA electrolytes.


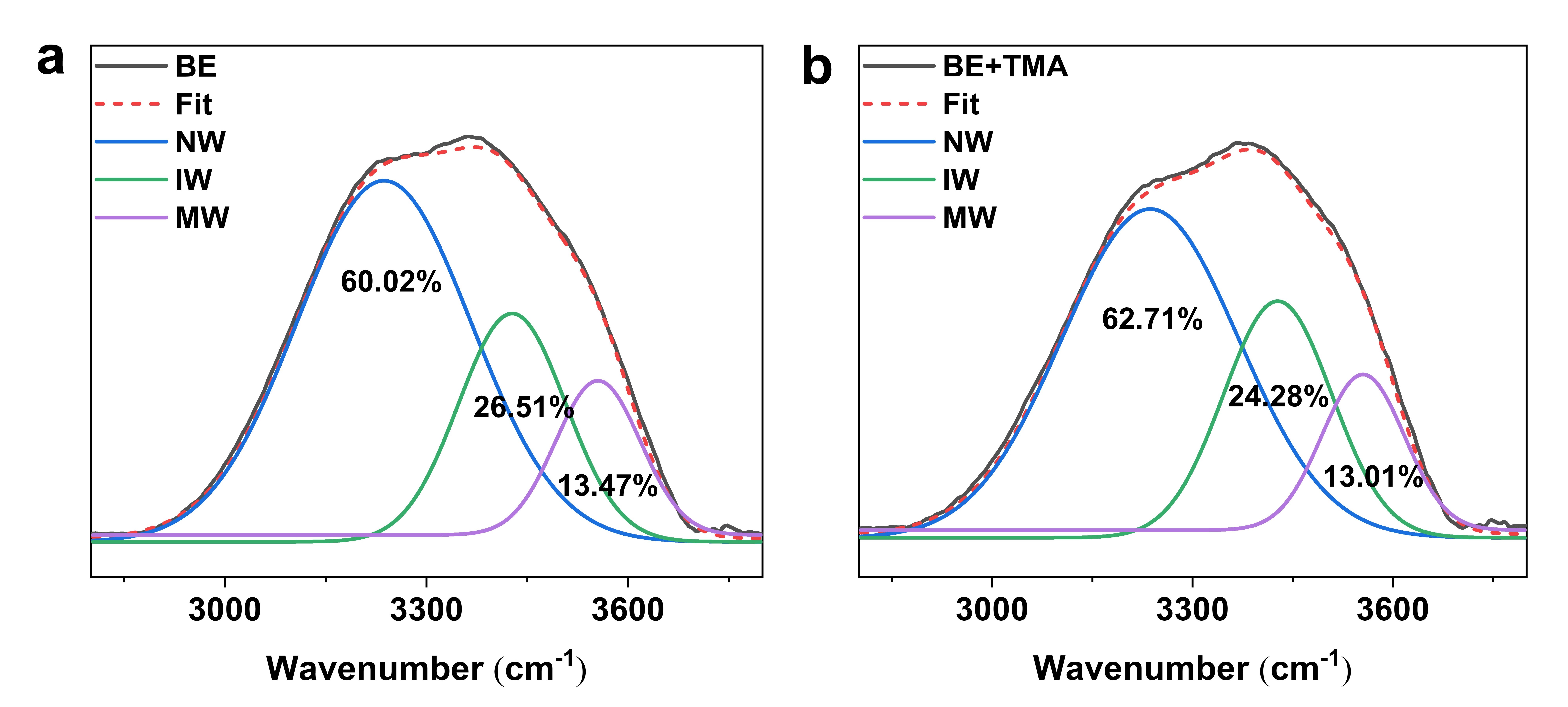


Figure S4. FTIR spectra of the H-O stretching band in (a) BE and (b) BE+TMA electrolytes.


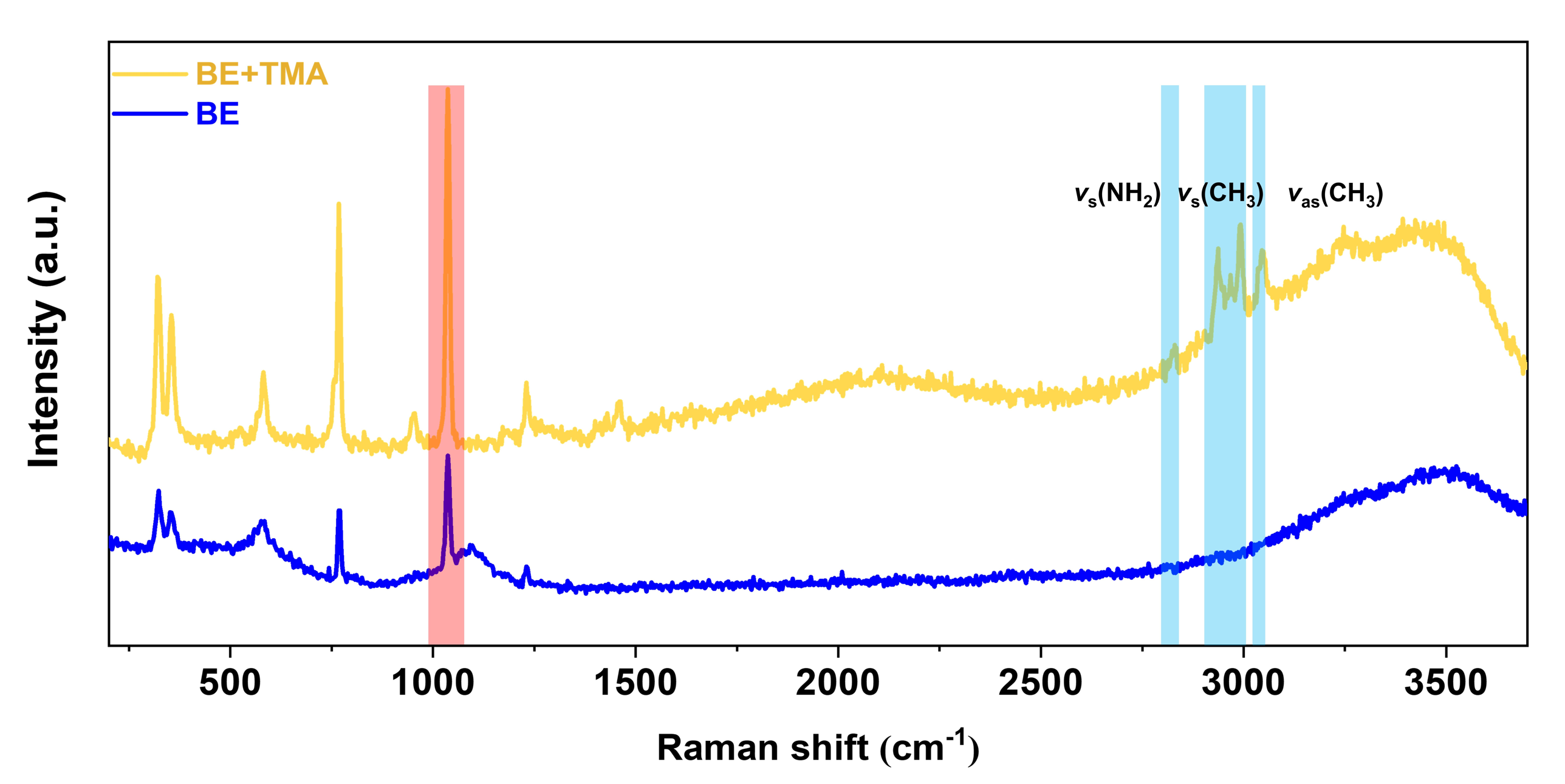


Figure S5. Full-range Raman spectra of BE and BE+TMA electrolytes.


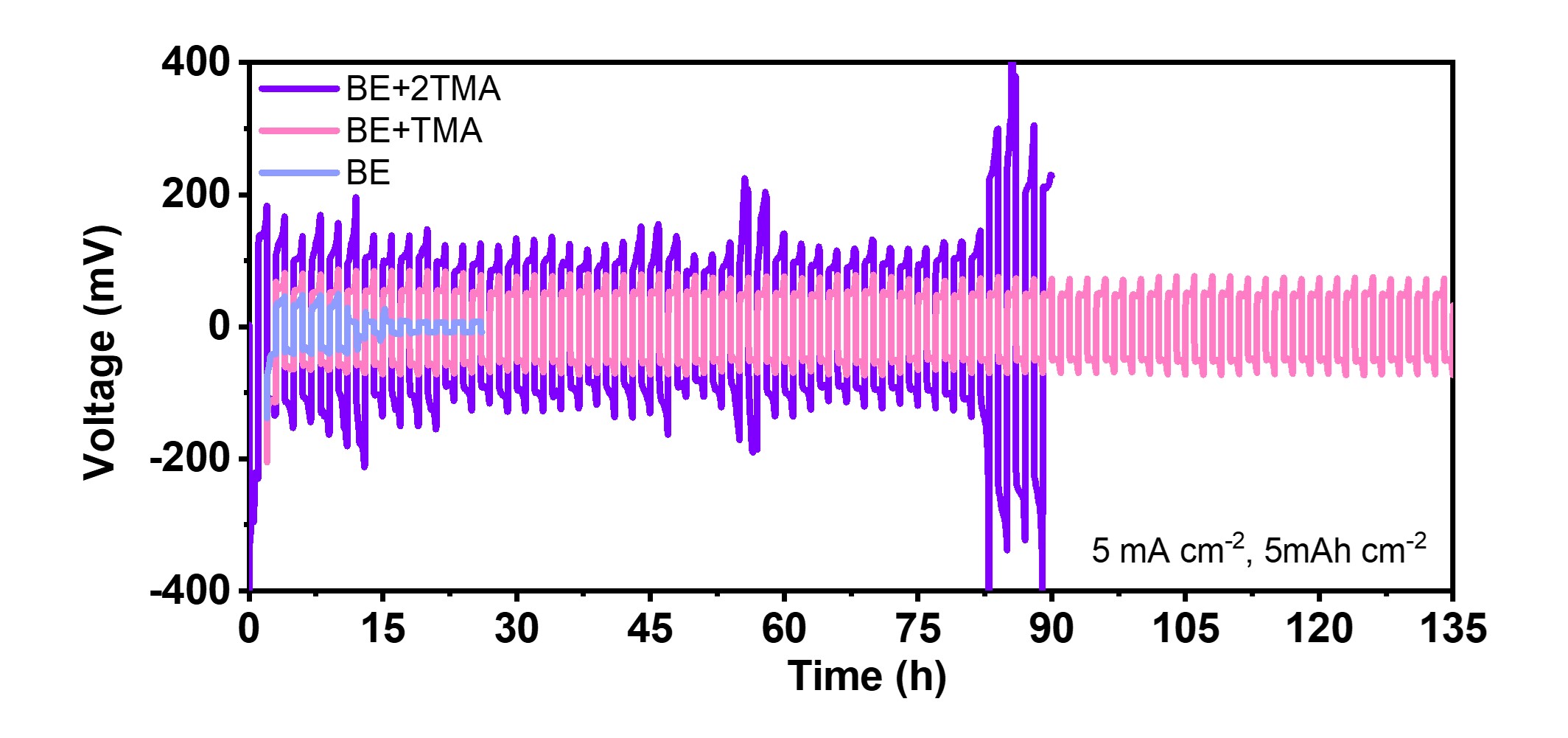


Figure S6. Long-term stripping/plating profiles of Zn||Zn symmetric cells at 5 mA cm^–2^ and 5 mAh cm^–2^ in BE, BE+TMA and BE+2TMA electrolytes.


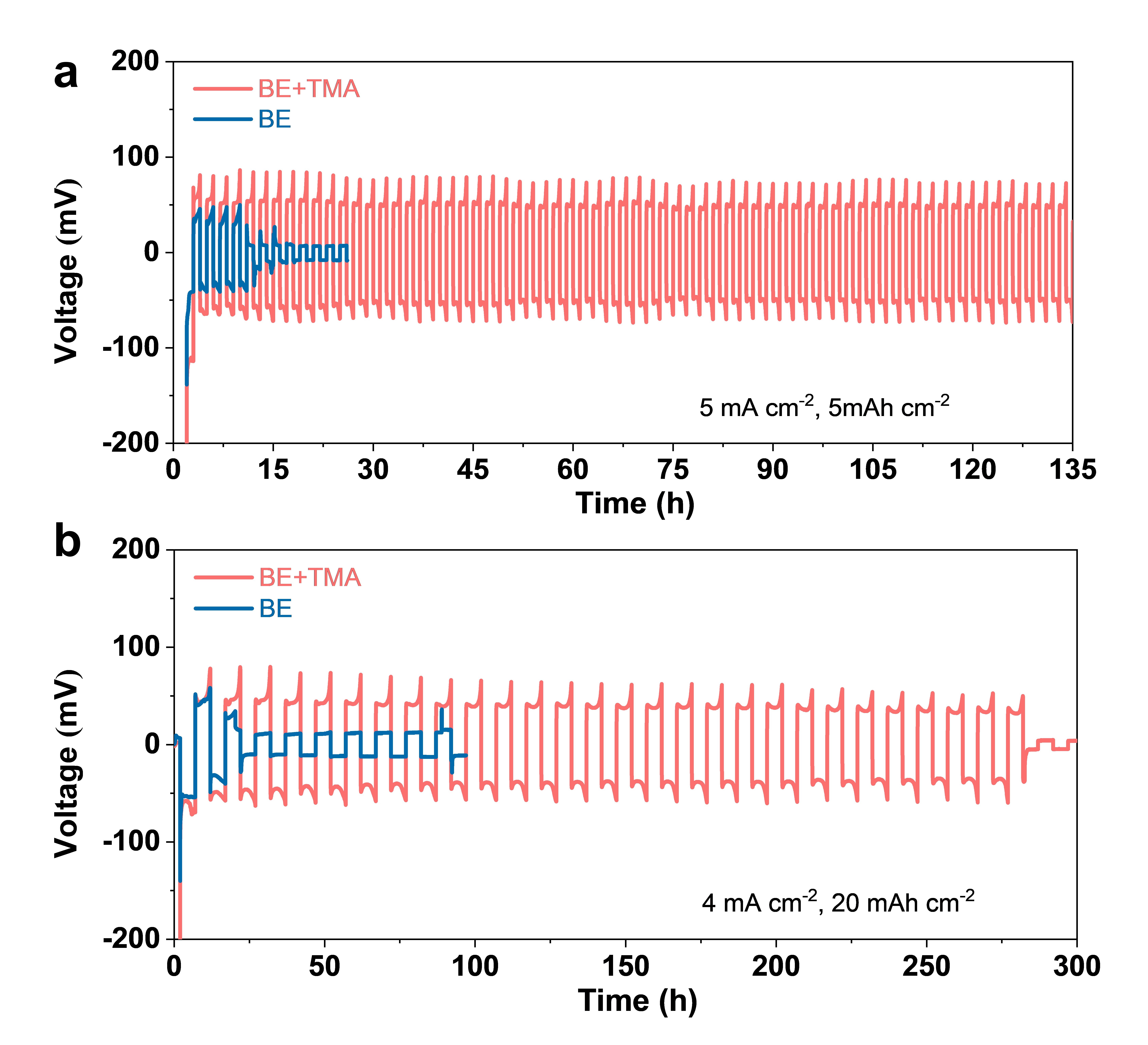


Figure S7. Long-term stripping/plating profiles of Zn||Zn symmetric cells (a) at 5 mA cm^–2^ and 5 mAh cm^–2^ and (b) at 4 mA cm^–2^ and 20 mAh cm^–2^.


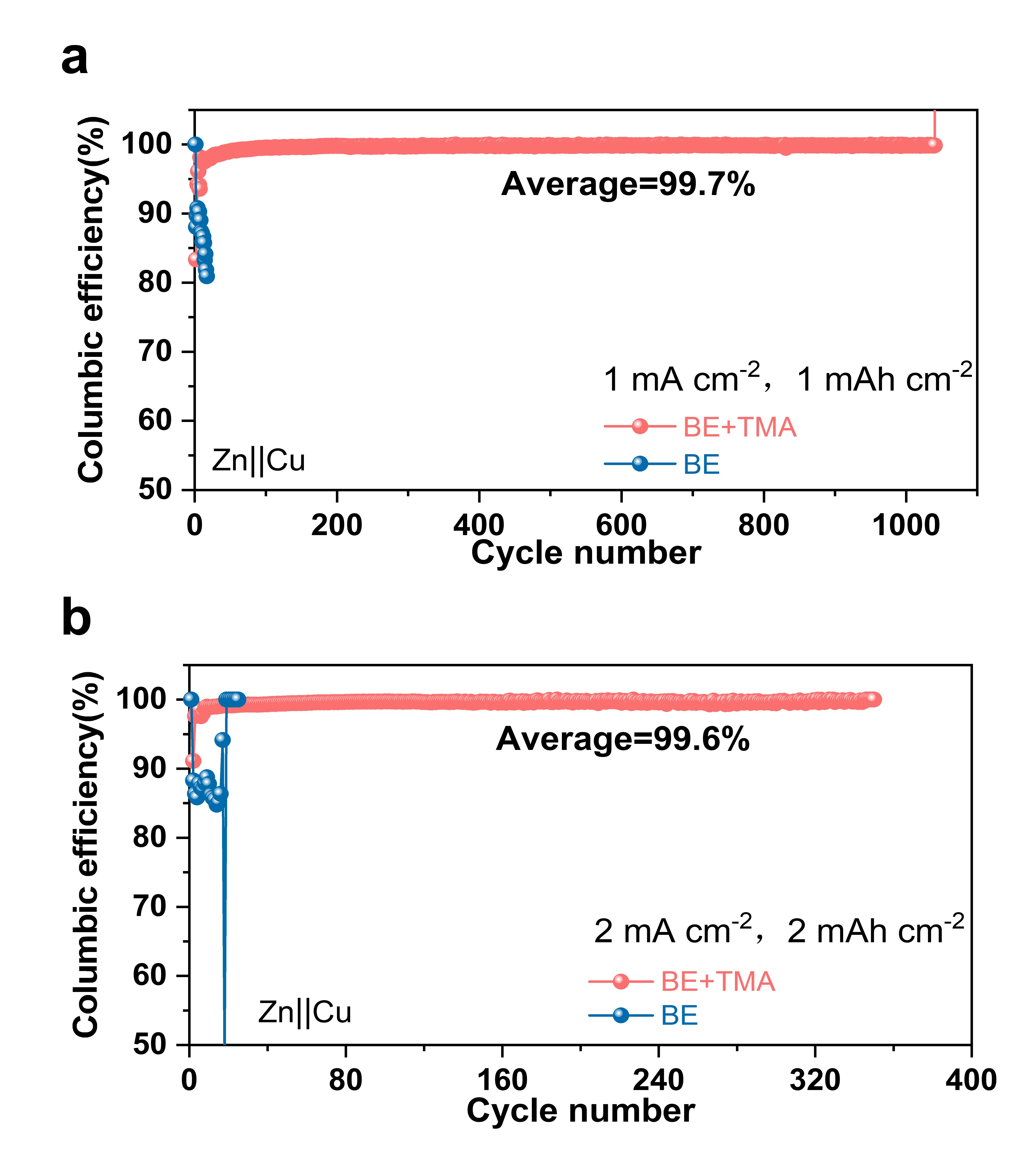


Figure S8. Coulombic efficiencies (CEs) of Zn plating/stripping in Zn||Cu cells at (a) 1 mA cm^–2^ and 1 mAh cm^–2^, and (b) 2 mA cm^–2^ and 2 mAh cm^–2^.


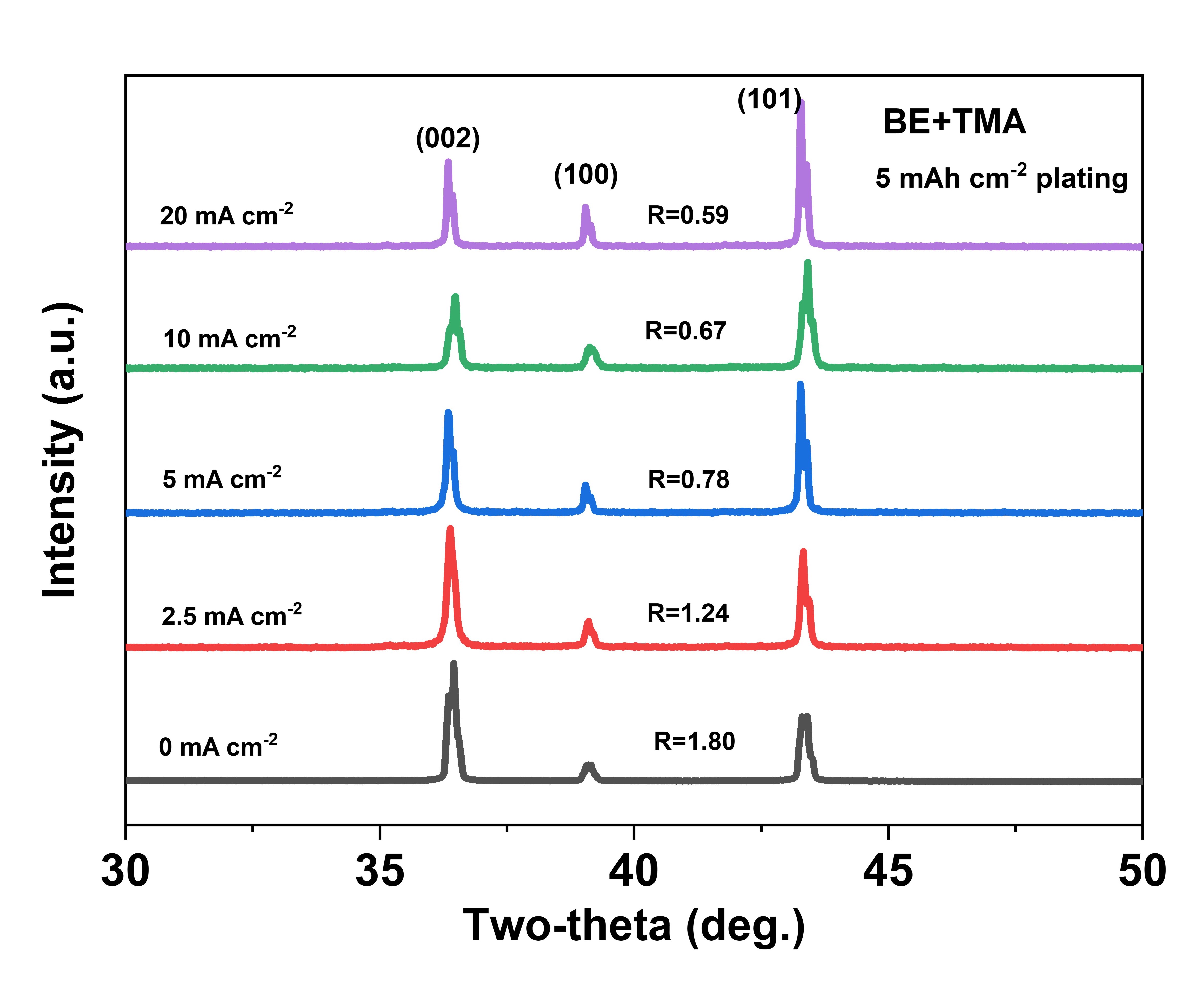


Figure S9. XRD patterns of the deposited Zn electrodes in BE+TMA at different current densities with a constant plated capacity of 5 mAh cm^–2^ (R = I_(002)_/I_(101)_).


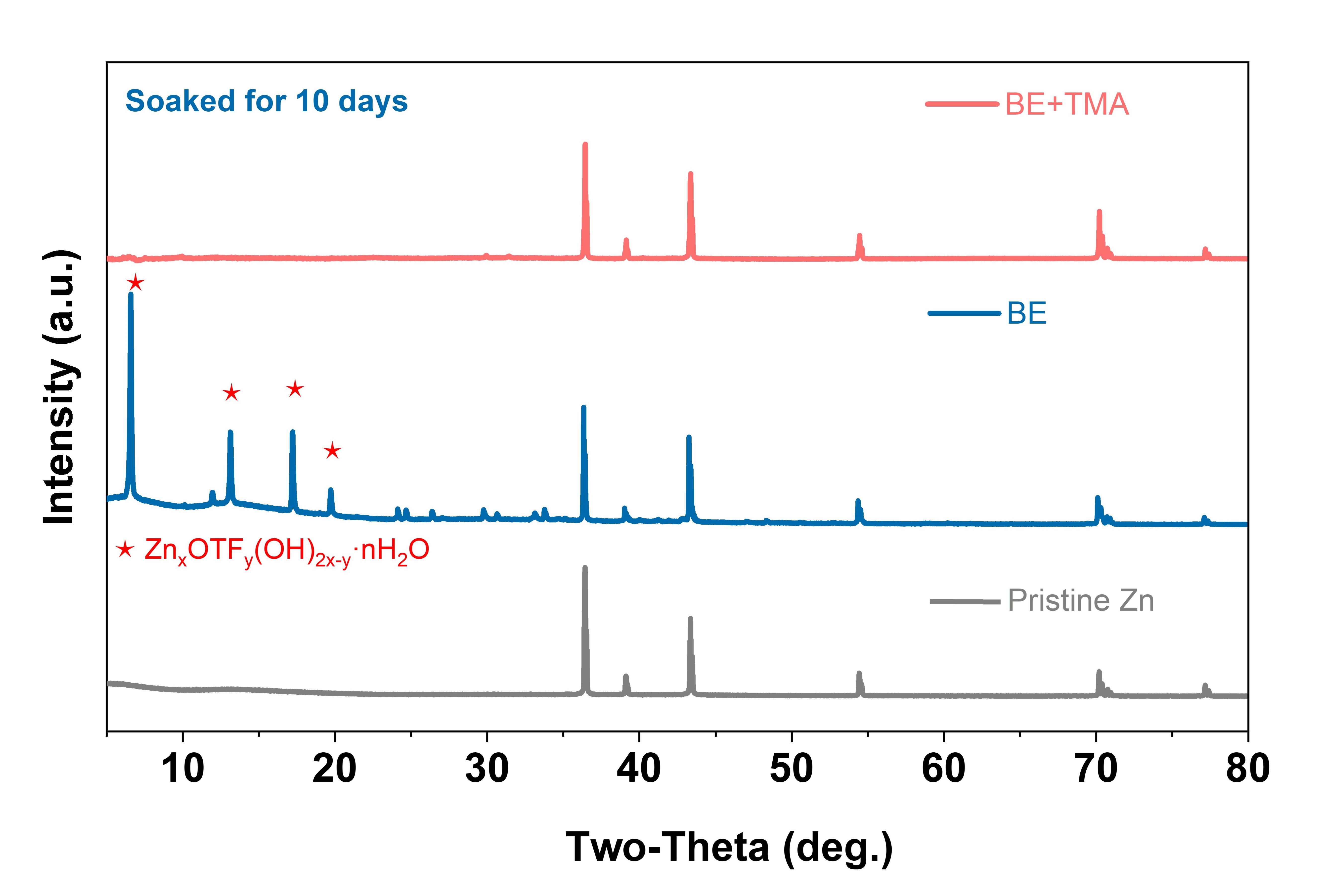


Figure S10. XRD patterns recorded on the Zn electrodes before and after soaking in aqueous electrolytes for 10 days.


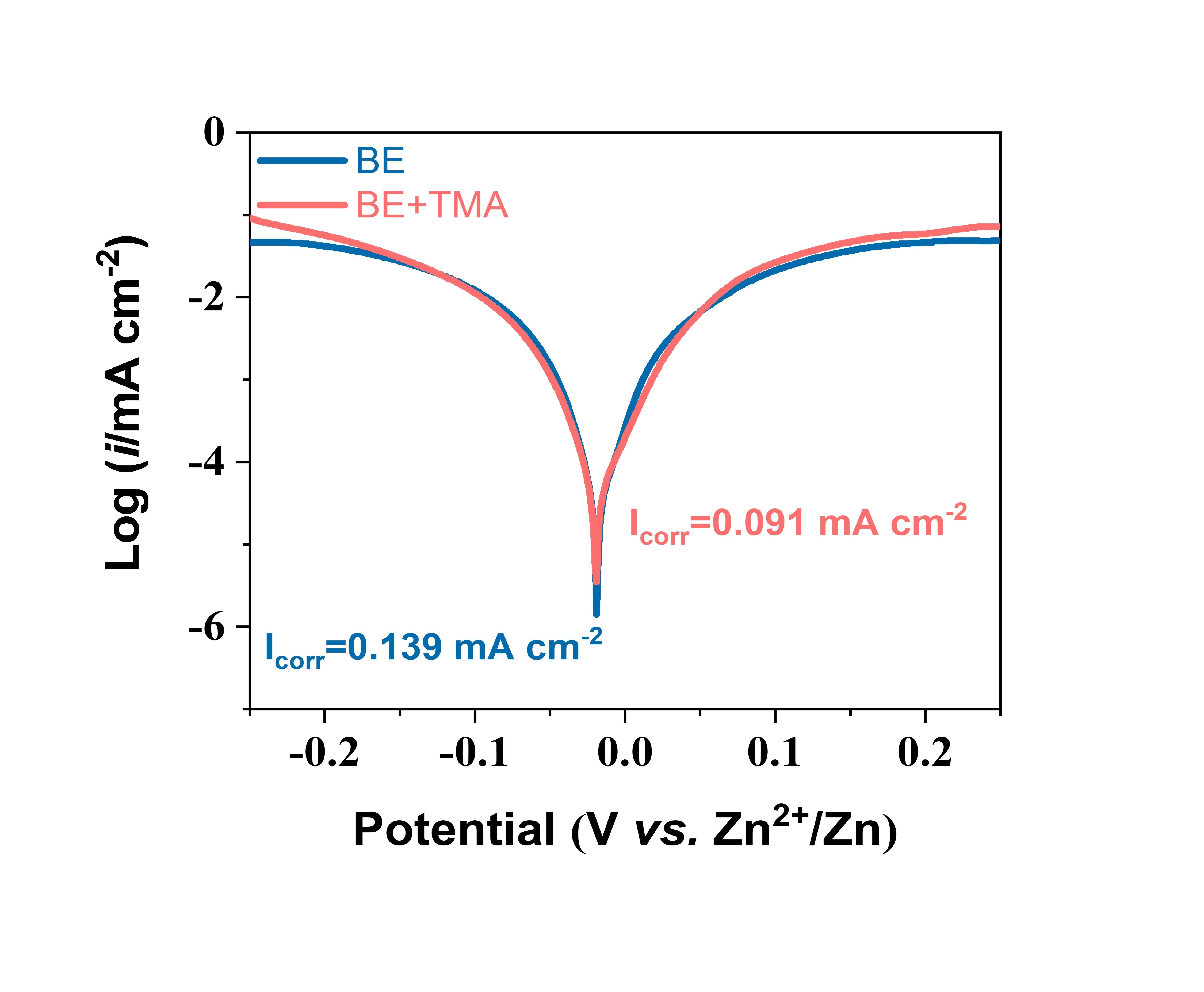


Figure S11. Tafel curves of BE and BE+TMA electrolytes.


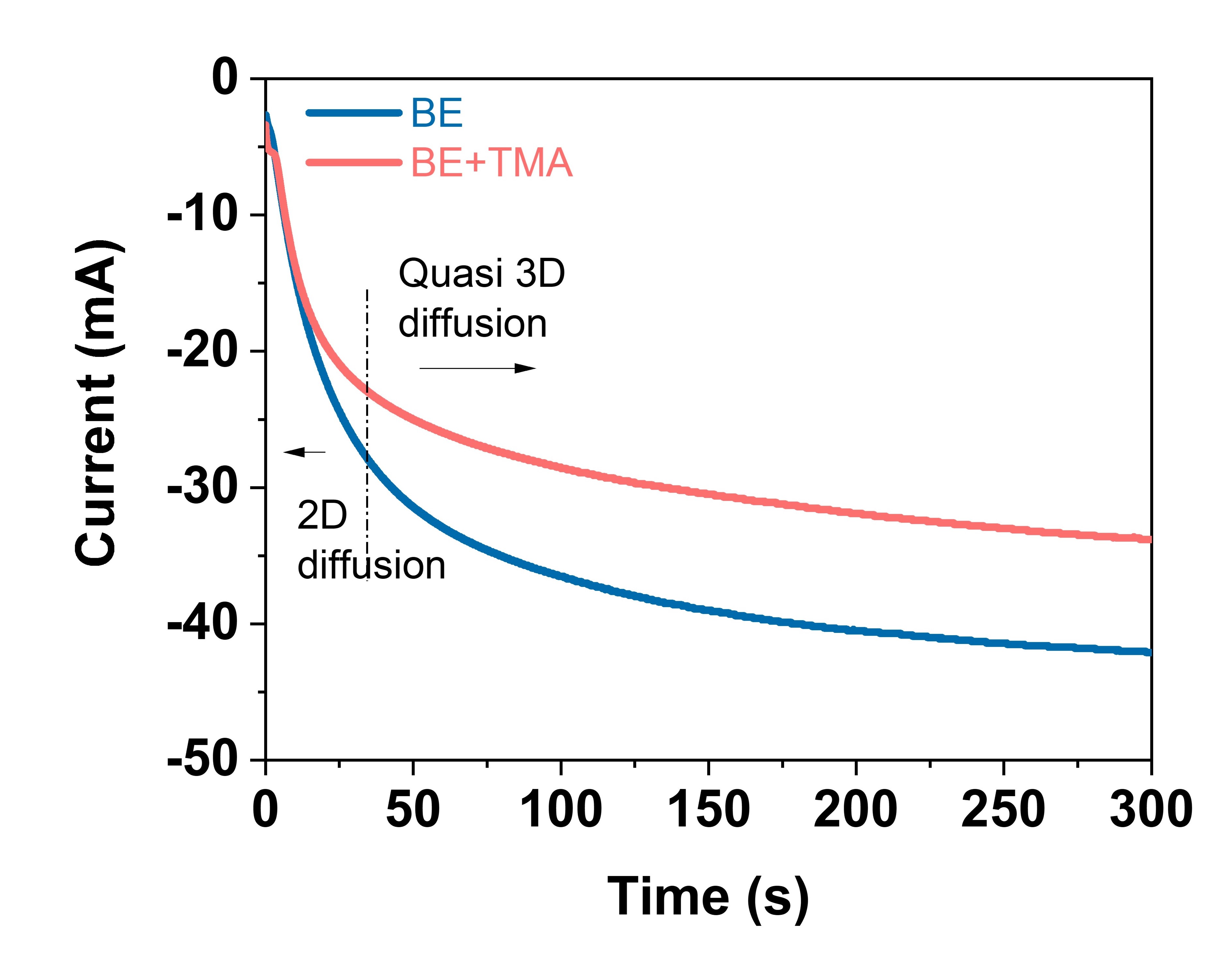


Figure S12. CA of Zn anodes tested in BE and BE+TMA electrolytes at a fixed overpotential of −150 mV.


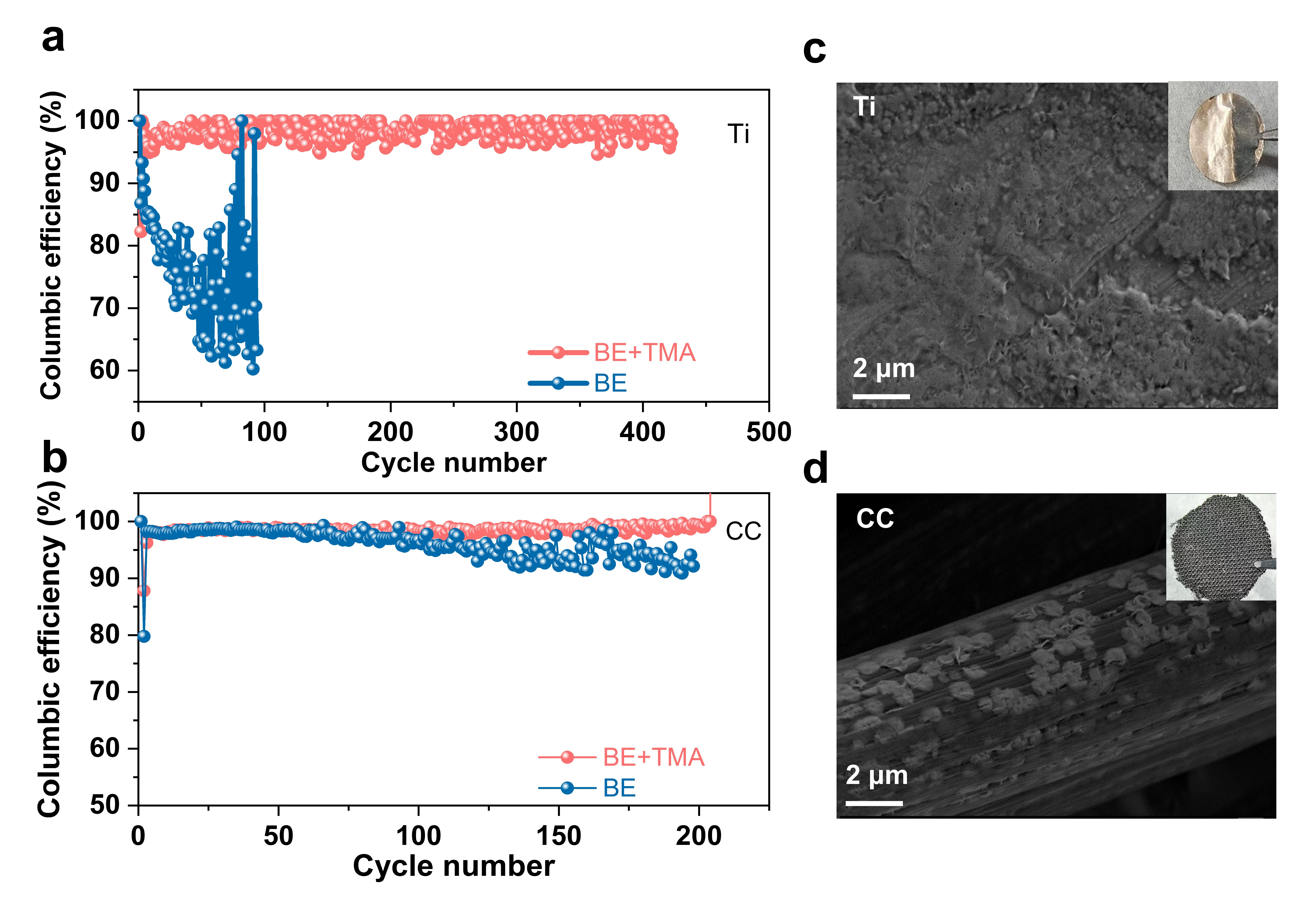


Figure S13. Coulombic efficiencies (CEs) of Zn plating/stripping and the recorded SEM images on the cycled substrates in (a, c) Zn||Ti and (b, d) Zn||carbon cloth (CC) cells at 0.5 mA cm^–2^ and 0.5 mAh cm^–2^. Insets show the photograph of the cycled Ti and CC electrodes.





Figure S14. Typical stripping-plating profiles of Zn||Cu symmetric cells in ZnSO_4_ and ZnSO_4_+TMA electrolytes.


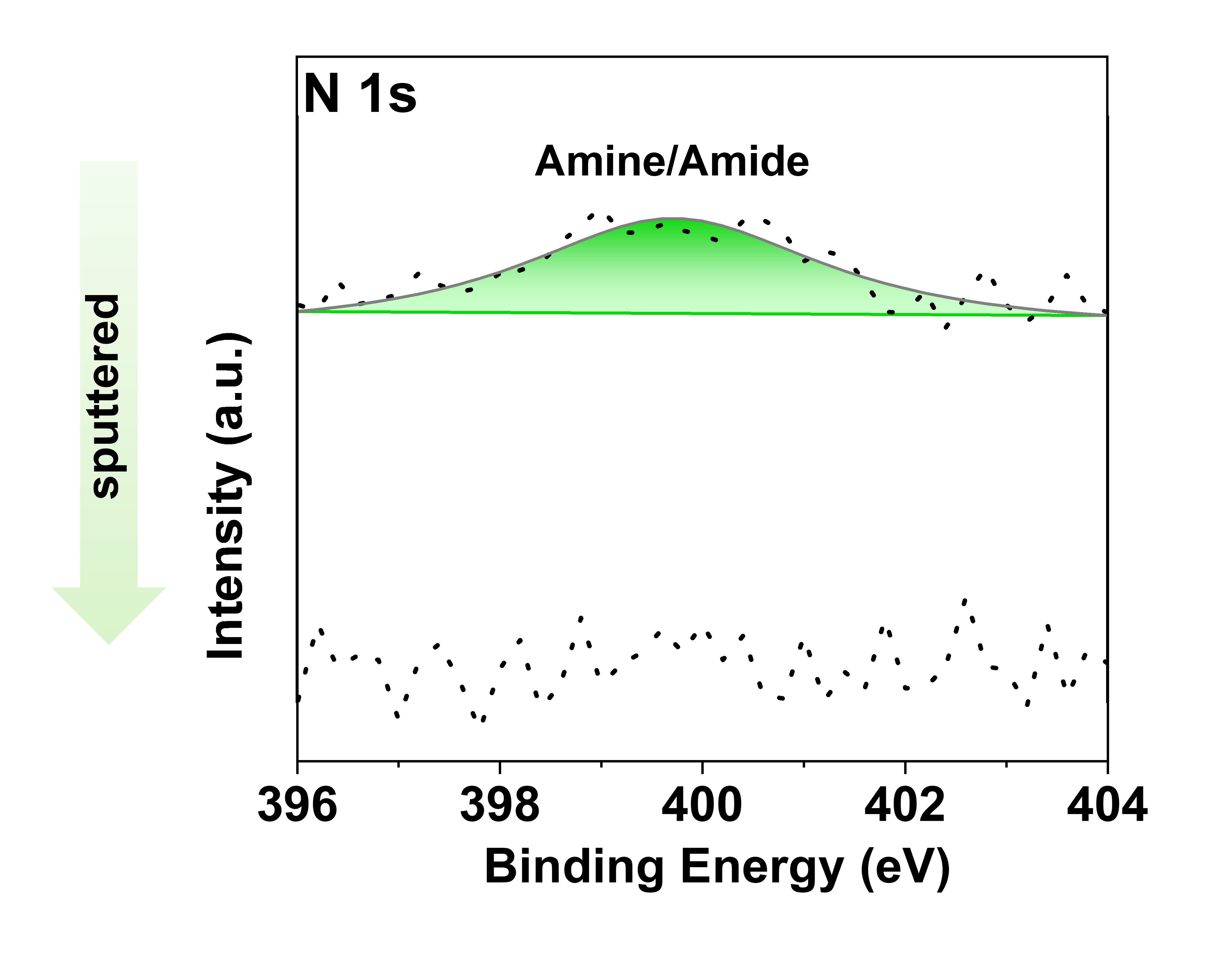


Figure S15. High resolution XPS N1s spectra of the cycled Zn electrodes.


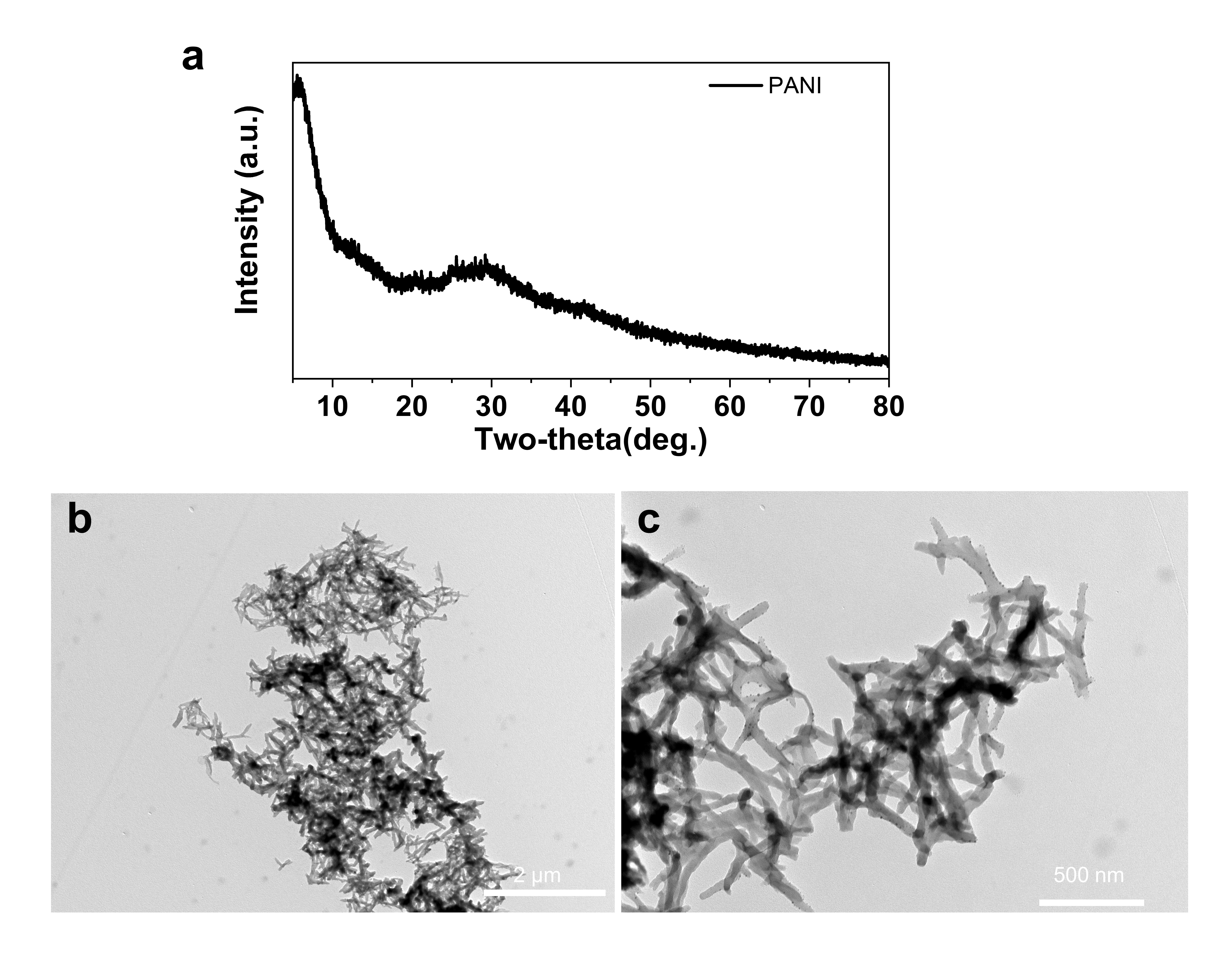


Figure S16. (a) XRD and (b, c) TEM images of as-synthesized PANI powders.


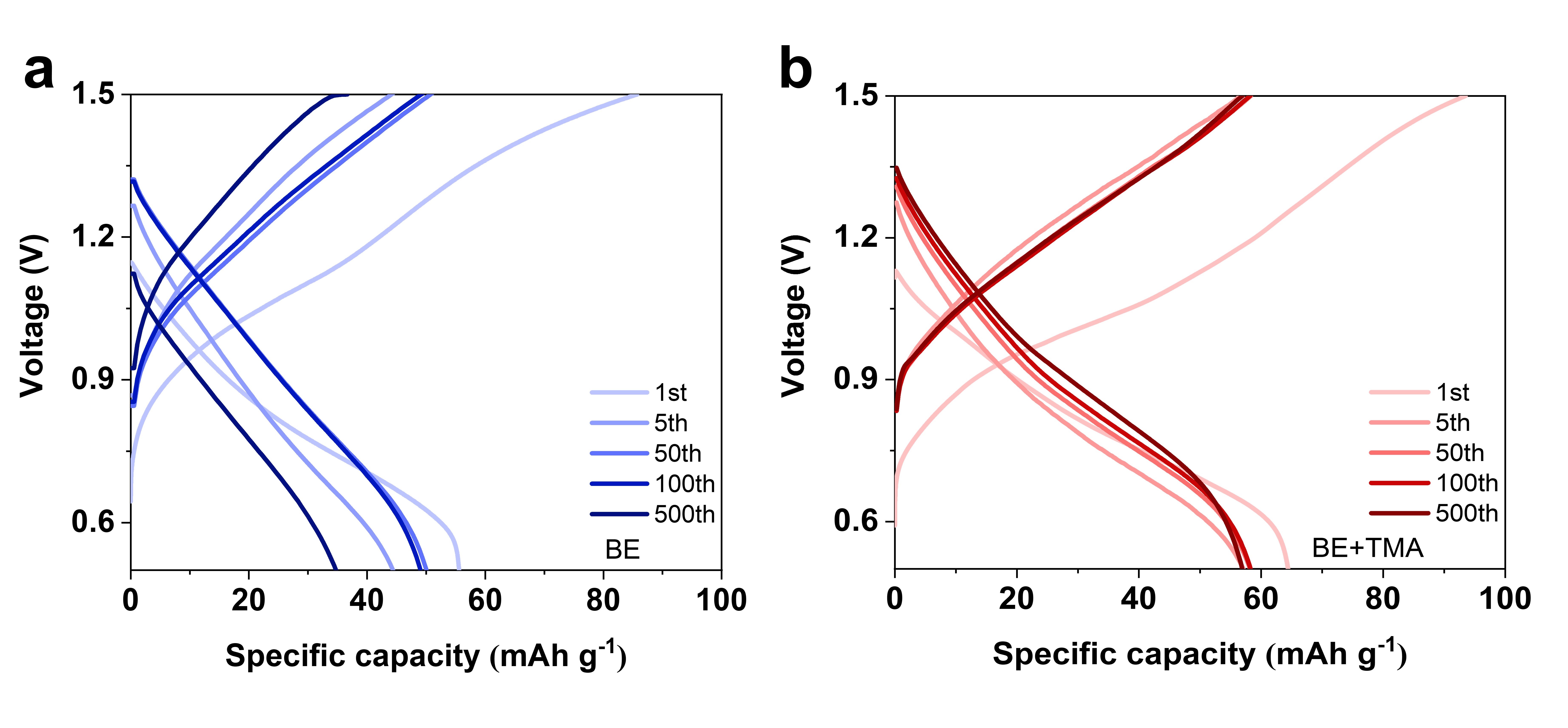


Figure S17. The selected voltage profiles of Zn||PANI full cells in BE and BE+TMA electrolytes.


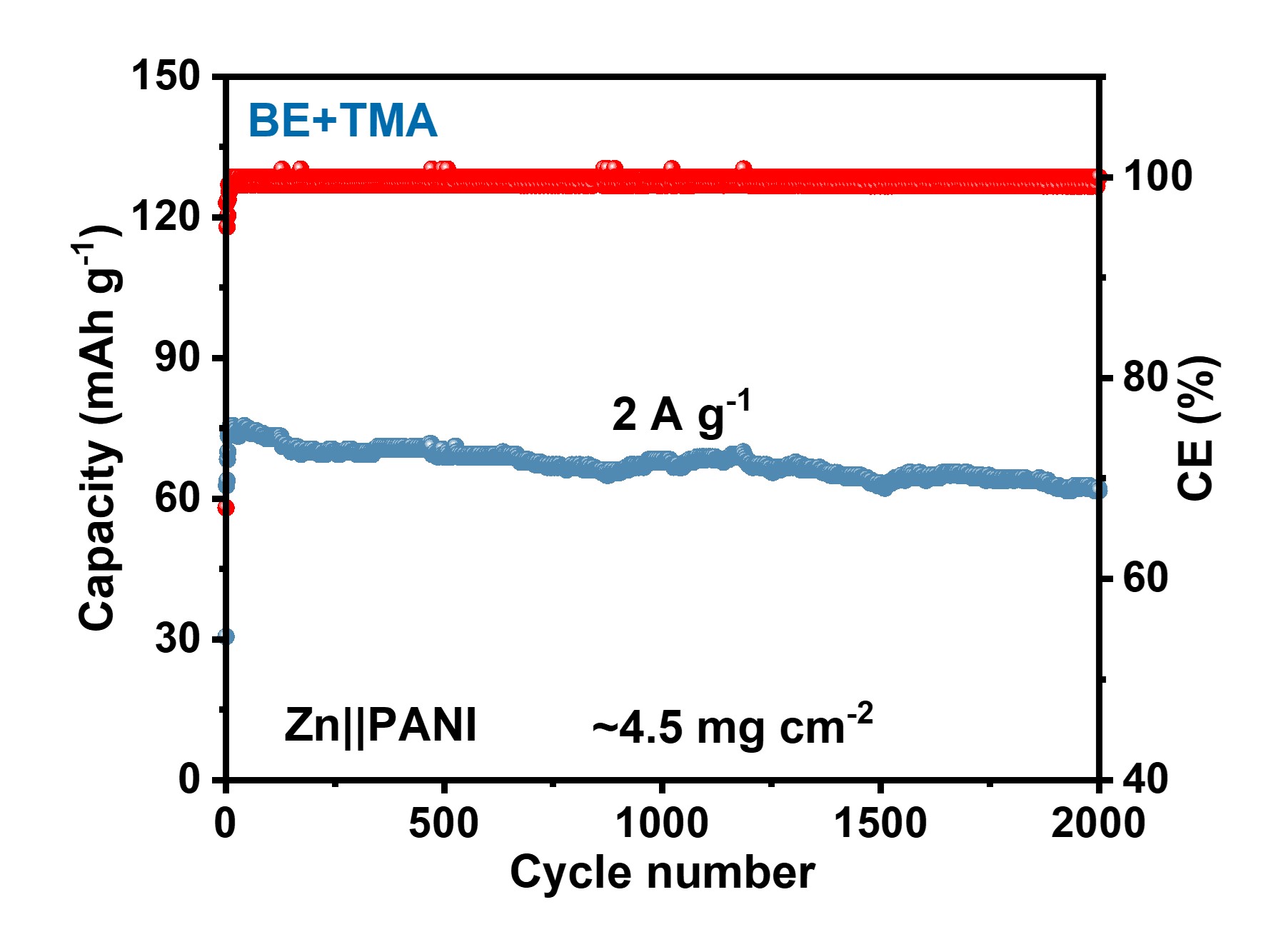


Figure S18. The cycling performance of Zn||PANI full cells in BE+TMA electrolyte with higher mass loading in cathode (mass loading: ~4.5 mg cm^-2^).


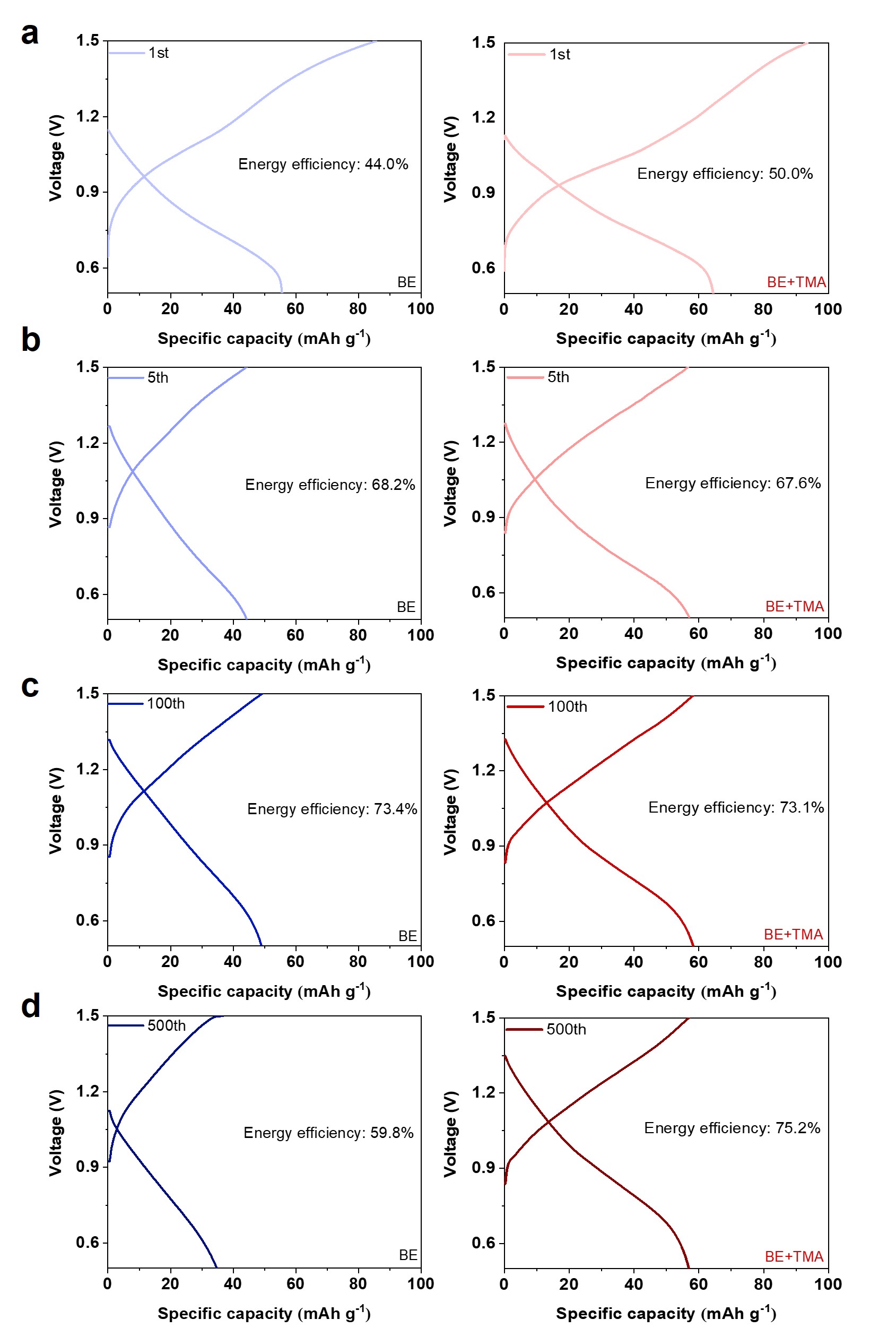


Figure S19. The selected voltage profiles of Zn||PANI cells at 2 A g^-1^ and the related energy efficiencies in BE and BE+TMA electrolytes at (a) 1^st^, (b) 5^th^, (c) 100^th^, and (d) 500^th^ cycles.


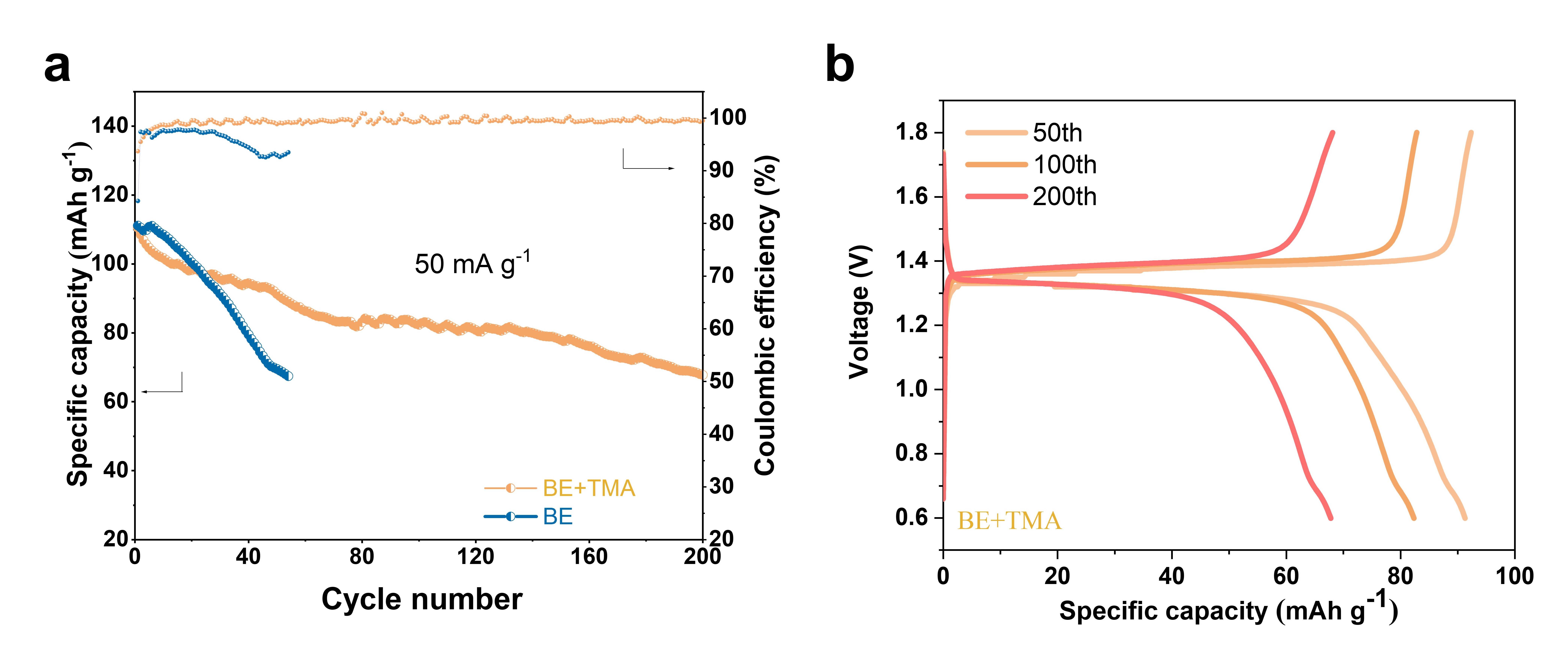


Figure S20. Electrochemical performance of Zn||NVP full cells in BE and BE+TMA electrolytes. (a) Long-term cycling performance and (b) selected GCD curves at 50 mA g^-1^.


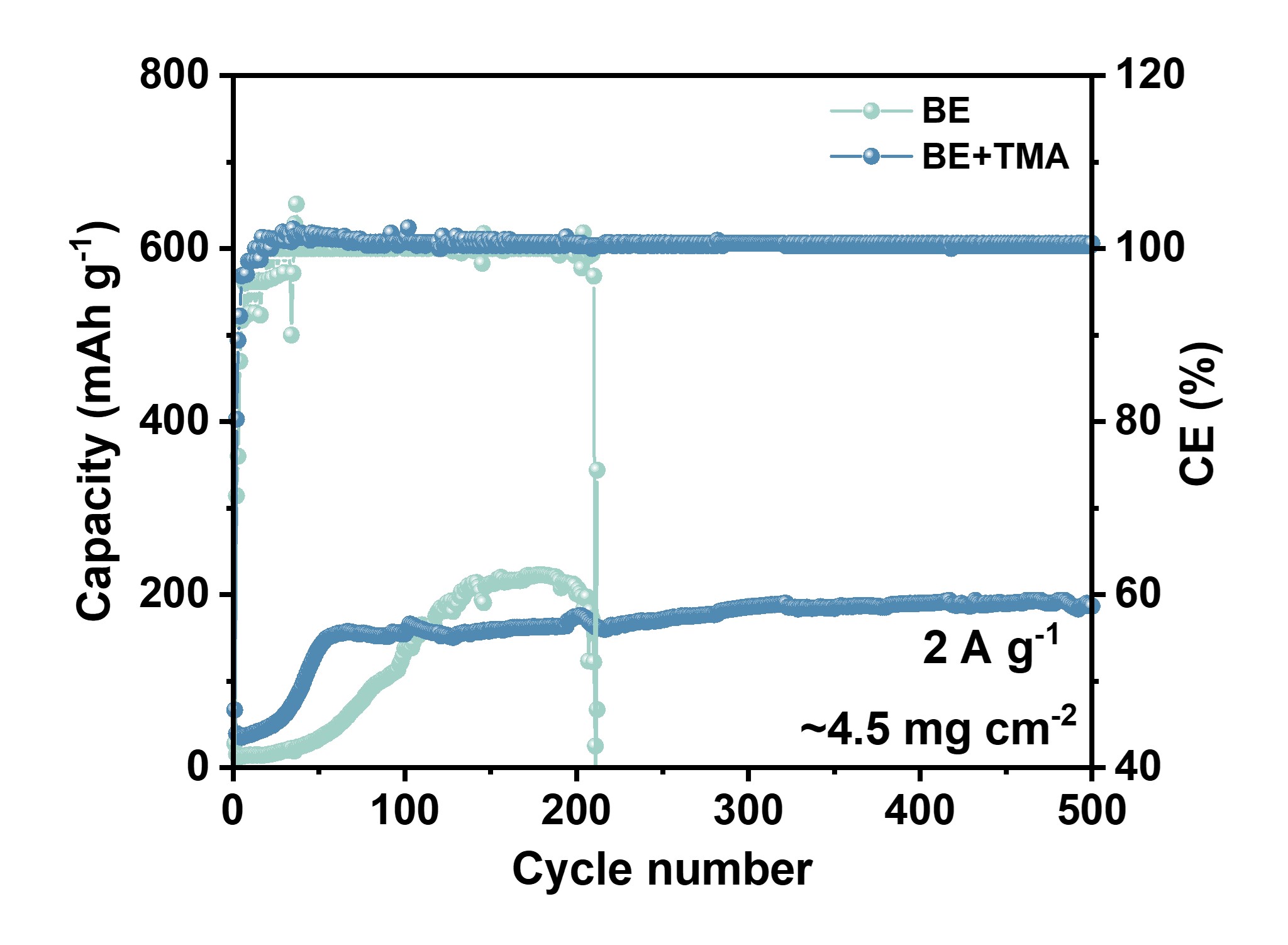


Figure S21. Long-term cycle stability test of Zn||V_2_O_5_ full cells at 2 A g^-1^ in BE and BE+TMA electrolytes with high mass loading in cathode (mass loading: ~4.5 mg cm^-2^).

Table S1. Cycle life and CE comparison with recently reported literatures utilizing different additives.

| Electrolyte  additive | CE (%) | Cycle (h) | Current density  (mA cm^-2^) | Areal capacity  (mAh cm^-2^) | Ref. |
| --- | --- | --- | --- | --- | --- |
| Methylammonium acetate | 98.7 | 700 | 0.5 | 0.5 | ^[12]^ |
| Cellulose | 97.3 | 830 | 140 | 1 | ^S[17]^ |
| Glucose | 97.2 | 200 | 1 | 0.5 | ^S[18]^ |
| Theanine | 99.6 | 850 | 5 | 1 | ^S[19]^ |
| Na4EDTA | 98.4 | 300 | 0.5 | 0.5 | ^S[20]^ |
| NH_4_H_2_PO_4_ | 99.4 | 1000 | 1 | 0.5 | ^S[21]^ |
| Tetraphenylporphyrin  tetrasulfonic acid | 99.8 | 2000 | 5 | 1 | ^S[22]^ |
| Glycerophosphorylcholine | 99.4 | 300 | 2 | 1 | ^S[23]^ |
| N, N'-Methylenebisacrylamide | 99.7 | 1300 | 1 | 1 | ^S[24]^ |
| Disodium lauryl phosphate | 98.2 | 550 | 1 | 1 | ^S[25]^ |
| EMIM[OAc] | 99.7 | 1000 | 1 | 0.5 | ^S[26]^ |
| L-carnitine | 98.1 | 1000 | 10 | 1 | ^S[27]^ |
| Sodium 3,3’-dithiodipropane sulfonate | 99.7 | 1200 | 1 | 1 | ^S[28]^ |
| Butanone | 99.9 | 300 | 1 | 1 | ^S[29]^ |
| Propylene carbonate | 99.8 | 150 | 1 | 0.5 | ^S[30]^ |
| Hexamethylenetetramine | 99.8 | 800 | 1 | 0.5 | ^S[31]^ |
| TMA | **99.8** | **2100** | **0.5** | **0.5** | **This work** |

***Supplementary References***

S[1] H. Zhang, B. Qin, D. Buchholz, S. Passerini, *ACS Applied Energy Materials* **2018**, 1, 6425.

S[2] K. Xiao, L. Yang, M. Peng, X. Jiang, T. Hu, K. Yuan, Y. Chen, *Small* **2024**, 20, 2306808.

S[3] C. I. Bayly, P. Cieplak, W. D. Cornell, P. A. Kollman, *J. Phys. Chem.* **1993**, 97, 10269.

S[4] J. M. Wang, W. Wang, P. A. Kollman, D. A. Case, *J. Mol. Graph. Model.* **2006**, 25, 247.

S[5] a) Y. Zhang, G. Wan, N. H. C. Lewis, J. Mars, S. E. Bone, H.-G. Steinrück, M. R. Lukatskaya, N. J. Weadock, M. Bajdich, O. Borodin, A. Tokmakoff, M. F. Toney, E. J. Maginn, *ACS Energy Lett.* **2021**, 6, 3458; b) E. Duboue-Dijon, P. Delcroix, H. Martinez-Seara, J. Hladilkova, P. Coufal, T. Krizek, P. Jungwirth, *J. Phys. Chem. B* **2018**, 122, 5640; c) L. Su, F. Lu, X. Liu, C. Wang, Y. Gao, S. Passerini, L. Zheng, X. Gao, *J. Power Sources* **2023**, 557, 232545.

S[6] H. J. C. Berendsen, J. R. Grigera, T. P. Straatsma, *J. Phys. Chem.* **1987**, 91, 6269.

S[7] L. Martinez, R. Andrade, E. G. Birgin, J. M. Martinez, *J Comput Chem* **2009**, 30, 2157.

S[8] B. Hess, C. Kutzner, D. van der Spoel, E. Lindahl, *J. Chem. Theory Comput.* **2008**, 4, 435.

S[9] H. J. C. Berendsen, J. P. M. Postma, W. F. Vangunsteren, A. Dinola, J. R. Haak, *J. Phys. Chem.* **1984**, 81, 3684.

S[10] G. Bussi, D. Donadio, M. Parrinello, *J. Chem. Phys.* **2007**, 126, 014101.

S[11] D. M. York, T. A. Darden, L. G. Pedersen, *J. Chem. Phys.* **1993**, 99, 8345.

S[12] B. Hess, H. Bekker, H. J. C. Berendsen, J. G. E. M. Fraaije, *J Comput Chem* **1997**, 18, 1463.

S[13] a) G. A. Petersson, A. Bennett, T. G. Tensfeldt, M. A. Al‐Laham, W. A. Shirley, J. Mantzaris, *The Journal of Chemical Physics* **1988**, 89, 2193; b) F. Weigend, R. Ahlrichs, *Physical Chemistry Chemical Physics* **2005**, 7, 3297.

S[14] A. V. Marenich, C. J. Cramer, D. G. Truhlar, *The Journal of Physical Chemistry B* **2009**, 113, 6378.

S[15] a) G. Kresse, J. Furthmüller, *Computational Materials Science* **1996**, 6, 15; b) G. Kresse, J. Furthmüller, *Physical Review B* **1996**, 54, 11169.

S[16] a) J. P. Perdew, K. Burke, M. Ernzerhof, *Physical Review Letters* **1996**, 77, 3865; b) J. P. Perdew, K. Burke, M. Ernzerhof, *Physical Review Letters* **1997**, 78, 1396.

S[17] Q. Wu, J. Huang, J. Zhang, S. Yang, Y. Li, F. Luo, Y. You, Y. Li, H. Xie, Y. Chen, *Angewandte Chemie International Edition* **2024**, 63, e202319051.

S[18] P. Sun, L. Ma, W. Zhou, M. Qiu, Z. Wang, D. Chao, W. Mai, *Angewandte Chemie International Edition* **2021**, 60, 18247.

S[19] N. Hu, W. Lv, W. Chen, H. Tang, X. Zhang, H. Qin, D. Huang, J. Zhu, Z. Chen, J. Xu, H. He, *Advanced Functional Materials* **2024**, 34, 2311773.

S[20] S.-J. Zhang, J. Hao, D. Luo, P.-F. Zhang, B. Zhang, K. Davey, Z. Lin, S.-Z. Qiao, *Advanced Energy Materials* **2021**, 11, 2102010.

S[21] W. Zhang, Y. Dai, R. Chen, Z. Xu, J. Li, W. Zong, H. Li, Z. Li, Z. Zhang, J. Zhu, F. Guo, X. Gao, Z. Du, J. Chen, T. Wang, G. He, I. Parkin, *Angewandte Chemie International Edition* **2023**, 62, e202212695.

S[22] J. Dong, L. Su, H. Peng, D. Wang, H. Zong, G. Wang, J. Yang, *Angewandte Chemie International Edition* **2024**, 63, e202401441.

S[23] H. Lyu, S. Zhao, C. Liao, G. Li, J. Zhi, F. Huang, *Advanced Materials* **2024**, 36, 2400976.

S[24] T. Yan, M. Tao, J. Liang, G. Zheng, B. Wu, L. Du, Z. Cui, H. Song, *Energy Storage Materials* **2024**, 65, 103190.

S[25] Q. Zhu, G. Sun, S. Qiao, D. Wang, Z. Cui, W. Zhang, J. Liu, *Advanced Materials* **2024**, 36, 2308577.

S[26] T. Xiao, J.-L. Yang, B. Zhang, J. Wu, J. Li, W. Mai, H. J. Fan, *Angewandte Chemie International Edition* **2024**, 63, e202318470.

S[27] H. Yu, D. Chen, X. Ni, P. Qing, C. Yan, W. Wei, J. Ma, X. Ji, Y. Chen, L. Chen, *Energy & Environmental Science* **2023**, 16, 2684.

S[28] Y. Lin, Z. Mai, H. Liang, Y. Li, G. Yang, C. Wang, *Energy & Environmental Science* **2023**, 16, 687.

S[29] X. Shi, J. Xie, J. Wang, S. Xie, Z. Yang, X. Lu, *Nature communications* **2024**, 15, 302.

S[30] F. Ming, Y. Zhu, G. Huang, A.-H. Emwas, H. Liang, Y. Cui, H. N. Alshareef, *Journal of the American Chemical Society* **2022**, 144, 7160.

S[31] H. Yu, D. Chen, Q. Li, C. Yan, Z. Jiang, L. Zhou, W. Wei, J. Ma, X. Ji, Y. Chen, L. Chen, *Advanced Energy Materials* **2023**, 13, 2300550.
